# Microphysical explanation of the RH‐dependent water affinity of biogenic organic aerosol and its importance for climate

**DOI:** 10.1002/2017GL073056

**Published:** 2017-05-21

**Authors:** N. Rastak, A. Pajunoja, J. C. Acosta Navarro, J. Ma, M. Song, D. G. Partridge, A. Kirkevåg, Y. Leong, W. W. Hu, N. F. Taylor, A. Lambe, K. Cerully, A. Bougiatioti, P. Liu, R. Krejci, T. Petäjä, C. Percival, P. Davidovits, D. R. Worsnop, A. M. L. Ekman, A. Nenes, S. Martin, J. L. Jimenez, D. R. Collins, D.O. Topping, A. K. Bertram, A. Zuend, A. Virtanen, I. Riipinen

**Affiliations:** ^1^ Department of Environmental Science and Analytical Chemistry (ACES) and Bolin Centre for Climate research Stockholm University Stockholm Sweden; ^2^ Department of Applied Physics University of Eastern Finland Kuopio Finland; ^3^ Department of Atmospheric and Oceanic Sciences McGill University Montreal Quebec Canada; ^4^ Department of Earth and Environmental Sciences Chonbuk National University Jeonju Republic of Korea; ^5^ Department of Chemistry University of British Columbia Vancouver British Columbia Canada; ^6^ Norwegian Meteorological Institute Oslo Norway; ^7^ Department of Civil and Environmental Engineering Rice University Houston Texas USA; ^8^ Cooperative Institute for Research in Environmental Sciences University of Colorado Boulder Colorado USA; ^9^ Department of Chemistry and Biochemistry University of Colorado Boudler Colorado USA; ^10^ Department of Atmospheric Sciences Texas A&M University College Station Texas USA; ^11^ Aerodyne Research Inc. Billerica Massachusetts USA; ^12^ Department of Chemistry Boston College Chestnut Hill Massachusetts USA; ^13^ School of Chemical and Biomolecular Engineering Georgia Institute of Technology Atlanta Georgia USA; ^14^ School of Earth and Atmospheric Sciences Georgia Institute of Technology Atlanta Georgia USA; ^15^ Institute for Environmental Research and Sustainable Development National Observatory of Athens Palea Penteli Greece; ^16^ School of Engineering and Applied Sciences Harvard University Cambridge Massachusetts USA; ^17^ Department of Physics University of Helsinki Helsinki Finland; ^18^ School of Earth and Environmental Sciences University of Manchester Manchester UK; ^19^ Department of Meteorology Stockholm University Stockholm Sweden; ^20^ Institute of Chemical Engineering Sciences, Foundation for Research and Technology‐Hellas Patras Greece; ^21^ National Centre for Atmospheric Science (NCAS) University of Manchester Manchester UK

**Keywords:** atmospheric aerosol, secondary organic aerosol, hygroscopicity, aerosol‐water interactions, aerosol‐climate interactions

## Abstract

A large fraction of atmospheric organic aerosol (OA) originates from natural emissions that are oxidized in the atmosphere to form secondary organic aerosol (SOA). Isoprene (IP) and monoterpenes (MT) are the most important precursors of SOA originating from forests. The climate impacts from OA are currently estimated through parameterizations of water uptake that drastically simplify the complexity of OA. We combine laboratory experiments, thermodynamic modeling, field observations, and climate modeling to (1) explain the molecular mechanisms behind RH‐dependent SOA water‐uptake with solubility and phase separation; (2) show that laboratory data on IP‐ and MT‐SOA hygroscopicity are representative of ambient data with corresponding OA source profiles; and (3) demonstrate the sensitivity of the modeled aerosol climate effect to assumed OA water affinity. We conclude that the commonly used single‐parameter hygroscopicity framework can introduce significant error when quantifying the climate effects of organic aerosol. The results highlight the need for better constraints on the overall global OA mass loadings and its molecular composition, including currently underexplored anthropogenic and marine OA sources.

## Key Points

- Phase separation effects explain differences in water affinity of biogenic secondary organic aerosol (SOA) at subsaturation and supersaturation
- Laboratory data for monoterpene and isoprene SOA are representative of field observations with corresponding gas‐phase organic profiles
- Importance of organic aerosol‐water interactions for global climate is governed by highly uncertain organic aerosol budgets

## 1 Introduction

An incomplete understanding of natural aerosols hampers the capability of the scientific community to quantify anthropogenic impacts on the global climate [*Stocker et al*., 2013; *Carslaw et al*., 2013]. Forests emit aerosol particles and their gaseous precursors, which influence atmospheric radiative transfer and cloud microphysics. The contribution of these effects to the global radiative budget is still highly uncertain and subject to intense debate [e.g., *Seinfeld et al*., 2016], in large part due to insufficient knowledge of the emissions and molecular processes involving atmospheric aerosol particles. Furthermore, creating simple yet robust and physicochemically sound descriptions of organic aerosol (OA) is essential for advancing the knowledge on aerosol‐climate interactions and quantifying the human influence on climate.

Aerosol particles originating from forest emissions contain primarily organic molecules, and a large fraction of this particulate mass is secondary, formed through the oxidation of volatile organic compounds (VOCs). Forests are one of the main sources of global OA, which makes up 20–90% of submicron particulate mass over the continents [*Jimenez et al*., 2009]. Different types of trees emit different mixtures of VOCs, resulting in differences in the SOA composition [*Guenther et al*., 2012; *Hu et al*., 2015]. While the SOA profile over coniferous forests is dominated by monoterpene (MT) oxidation products, the SOA from broad‐leaved trees are dominated by compounds formed through the photooxidation of isoprene (IP). The molecular composition of biogenic SOA is complex [*Goldstein and Galbally*, 2007] and dynamic due to chemical transformation in the atmosphere over timescales ranging from minutes to days [*Jimenez et al*., 2009]. This atmospheric processing modifies the SOA composition [*Hu et al*., 2015], polarity, carbon number, oxidation state [*Kroll et al*., 2011], volatility [*Bilde et al*., 2015], and phase state [*Virtanen et al*., 2010].

Interactions of aerosol particles with water vapor are important for determining the behavior and effects of atmospheric aerosol, given the comparably high abundance of water in the air and its importance for various processes in the Earth system. Specifically, hygroscopicity is defined as the extent to which an aerosol particle takes up water when exposed to a given relative humidity (RH). If RH is increased above water saturation (RH > 100%), the particles may act as cloud condensation nuclei (CCN) [*Kohler*, 1936; *Raymond and Pandis*, 2002] and form new cloud droplets influencing the radiative properties and lifetime of clouds [*Lohmann and Feichter*, 2005]. Aerosol hygroscopicity and CCN activation are often represented with a single, semi‐empirical hygroscopicity parameter *κ* at both subsaturated and supersaturated RH [*Petters and Kreidenweis*, 2007]. Laboratory data indicate, however, that the *κ* parameters measured for SOA (*κ*_SOA_) at subsaturated (RH < 100%) and supersaturated (RH > 100%) conditions can vary substantially [*Prenni et al*., 2007; *Wex et al*., 2009; *Pajunoja et al*., 2015; *Hodas et al*., 2016]. On the other hand, many current climate models represent the organic aerosol fraction with one to two surrogate species with specific molecular properties [*Tsigaridis et al*., 2014]. This is disparate to the detailed model description of the hygroscopicity and CCN activation of inorganic aerosols [*Baklanov et al.*, 2014].

In this work we present a microphysical explanation of the behavior of the RH‐dependent water affinity of biogenic SOA produced from IP and α‐pinene (as a representative MT species) oxidation. The proposed mechanistic picture is based on the synthesis and interpretation of a comprehensive set of laboratory and field data using thermodynamic models that allow accounting for differences in the aerosol composition. Finally, we put the results into a larger context through studying the sensitivity of climate forcing reproduced by two state‐of‐the‐art climate models to the water affinity of OA.

## 2 Materials and Methods

We used two theoretical approaches with varying level of complexity to describe the RH‐dependent water uptake and CCN activation behavior of the IP‐ and MT‐derived SOA. The first, more simplistic, approach was based on a description of limited solubility of the SOA components using solubility distributions [*Hilal et al*., 1995; *Riipinen et al*., 2015] coupled with treatment of adsorption using the Frenkel‐Halsey‐Hill adsorption theory [*Frenkel*, 1946; *Halsey*, 1948; *McDonald*, 1964; *Jiusto and Kocmond*, 1968; *Hill*, 1949; *Sorjamaa and Laaksonen*, 2007; *Kumar et al*., 2009, 2011a, 2011b] (see supporting information (SI) for details). The limited solubility in water is a manifestation of nonideality [*Prausnitz et al*., 1964]. In our first‐order approximations, this was the only consequence of nonideality taken into account in the water‐uptake calculations (see section 3 in SI) predicted by the SPARC prediction tool [*Hilal et al*., 1995; *Wania et al.*, 2014; *Riipinen et al*., 2015], assuming that water and organic phase otherwise behave as ideal mixtures, yielding *Γ*_w_ = 1. To explore the nonideal behavior further, we used the multiphase system online property prediction (UManSysProp) for calculating *Γ*_w_ in organic solution droplets. UManSysProp (http://vm‐woody009.itservices.manchester.ac.uk/index) is an online application developed for calculating the properties of individual molecules, mixtures (organic, inorganic, or mixed organic‐inorganic), and aerosol particles. For calculating activity coefficients in aqueous solutions, the Aerosol Inorganic‐Organic Mixtures Functional groups Activity Coefficients (AIOMFAC) model [*Zuend et al*., 2010; *Zuend and Seinfeld*, 2012] is applied within UManSysProp. In the second, more comprehensive approach, a gas‐particle partitioning model based on AIOMFAC and the pure compound liquid‐state saturation vapor pressure prediction model EVAPORATION (Estimation of Vapour Pressure of Organics, Accounting for Temperature, Intramolecular, and Non‐additivity effects) [*Compernolle et al*., 2011], available online at http://tropo.aeronomie.be/models/evaporation_run.htm, was used. This equilibrium gas‐particle partitioning model includes the prediction of a potential liquid‐liquid phase separation (i.e., a liquid‐liquid equilibrium state) in the liquid particle mixture [*Zuend and Seinfeld*, 2013]. The model was run similar to the case studies by *Zuend et al.* [2010] to account for the concurrent water uptake and partitioning of semivolatile organic compounds contributing to the effective hygroscopic growth at a given RH [*Surratt et al*., 2010; *Kristensen et al*., 2013; *Zhang et al*., 2015].

To visually observe the phase state behavior of the two SOA types, optical images of micrometer‐scale SOA particles were used [*Bertram et al*., 2011]. Such images for SOA derived from ozonolysis of α‐pinene have been reported previously [*Renbaum‐Wolff et al*., 2016]. Optical images of SOA particles derived from photooxidation of isoprene are described here: Isoprene‐derived SOA was generated by the photooxidation of isoprene in an oxidation flow reactor [*Kang et al*., 2007; *Lambe et al*., 2011; *Liu et al*., 2015]. Table S1 lists the experimental conditions for SOA production. At the exit of the oxidation flow reactor, particles were collected on a hydrophobic glass slide using a single stage impactor or electrostatic precipitator [*Renbaum‐Wolff et al*., 2016]. After collection, the hydrophobic glass slide was inserted into a temperature and relative humidity controlled flow cell coupled to an optical microscope (Zess Axiotech, 50× objective). RH was controlled within the cell by varying the ratio of a dry and humidified N_2_ flow with the total flow rate of ~1200 sccm. The RH was measured using a hygrometer with a chilled mirror sensor (General Eastern, Canada), which was calibrated using deliquescence RH for pure ammonium sulfate particles (uncertainty of the RH: ±1.0%). After the glass slide containing the SOA particles was inserted into the flow cell, the SOA particles were equilibrated at ~100% RH for 15 min, and then the RH was scanned from ~100% to ~0% RH and subsequently ~0% to ~100% RH at a rate of 0.1–0.5% RH min^−1^. During the humidity cycles, optical images of the SOA particles were recorded every 5–10 s using a CCD camera. All experiments were performed at constant temperature of 290 ± 1 K. From the optical images, the presence of one or multiple phases could be identified.

To investigate the RH‐dependent water uptake and CCN behavior of ultrafine SOA particles, we used the laboratory data set from *Pajunoja et al.* [2015] for particles formed from the photooxidation of isoprene (C_5_H_8_, IP) or ozonolysis of α‐pinene (C_10_H_16_, MT). In both cases (MT and IP), SOA was formed in a continuous flow Potential Aerosol Mass flow reactor [*Kang et al*., 2007; *Liu et al*., 2015]. The reactor was operated in continuous flow mode with a mean residence time of approximately 100 s and a humidified carrier gas (RH ~ 30%) containing a synthetic mixture of N_2_ and O_2_. Trace levels of SOA precursors (isoprene or α‐pinene) were mixed with the carrier gas at the inlet of the reactor. SOA precursors react with O_3_ or OH radicals inside the reactor, after which low‐vapor pressure oxidation products homogenously nucleated to form SOA particles. The sample flow was then dried with a diffusion dryer prior to composition and hygroscopicity measurements with a high‐resolution time‐of‐flight aerosol mass spectrometer (HR‐ToF‐AMS; Aerodyne Research, Inc.) [*DeCarlo et al*., 2006], a Hygroscopic Tandem Differential Mobility Analyzer (HTDMA), and a Cloud Condensation Nuclei counter (CCNc; Droplet Measurement Technologies) [*Brechtel and Kreidenweis*, 2000; *Roberts and Nenes*, 2005; *Paramonov et al*., 2013; *Guo et al*., 2015]. In *Pajunoja et al.* [2015], the elemental oxygen‐to‐carbon ratio (O:C) = 0.45 of α‐pinene SOA particles was calculated from HR‐ToF‐AMS measurements using the Aiken analysis method [*Aiken et al*., 2008], and O:C = 0.86 of IP‐SOA was calculated using the method introduced in *Chen et al.* [2011]. Here we apply the revised elemental analysis method [*Canagaratna et al*., 2015] to update O:C of α‐pinene SOA from 0.45 to 0.56. The hygroscopic growth factors (HGF) of the SOA particles were measured at varied subsaturated conditions (five RH steps) with the HTDMA, and the cloud activation properties were measured at liquid water supersaturation (SS = 0.1–1.0%) with the CCNc. An initial dry particle mobility diameter of 100 nm was selected in both instruments.

Ambient measurements used in this study comprised comprehensive measurement campaigns carried out in the Southeastern US, Centreville, Alabama (32.90289°N, 87.24968°W, 126 m above sea level (asl)) [*Hu et al*., 2015; *Xu et al*., 2015], and in Northern Europe, Finland, Hyytiälä (61.84524°N, 24.28883°E, 181 m asl) in the summer of 2013. Both sites are rural environments with dominance of biogenic emission sources. The airborne particle population was characterized by size and composition with a Differential Mobility Particle Sizer and HR‐ToF‐AMS (see SI for details), respectively. Moreover, the hygroscopic properties of the particles were measured both at subsaturated and supersaturated conditions with HTDMA and CCNc instruments, respectively. Humidity control in the HTDMA and CCNc were similar to the laboratory measurements, but the water saturations in the setups were fixed to RH = 90% and SS = 0.2%, respectively. Only time periods with OA mass fraction (based on the AMS analysis) of the particles *f*_org_ ≥ 0.6 were used in the analysis. Similar analysis methods of hygroscopicity were used as in previous studies [*Pajunoja et al*., 2016; *Hong et al*., 2014; *Cerully et al*., 2015]. The mixture's effective hygroscopicity parameter *κ* was derived for the organic‐dominated (*f*_org_ ≥ 0.6) ambient particles (*κ*_HGF_ from HTDMA and *κ*_CCN_ from CCNc) and also for the organic fraction of the particles (*κ*_HGF,org_ and *κ*_CCN,org_). *κ* for the organic fraction was derived using a mixing rule [*Petters and Kreidenweis*, 2007] and by (1) assuming AMS PM_1_ mass fractions to be representative for 100 nm particles; (2) categorizing inorganic fraction into sulfuric acid (SA), ammonium sulfate (AS), and ammonium bisulfate (ABS) [*Nenes et al*., 1998]; and (3) using published *κ* and density values for the inorganic species [*Pajunoja et al*., 2016]. Effect of inlet RH on the amount of residual water (due to highly hygroscopic SA) in the “dry” particle phase was taken into account. In the Alabama measurements, the inlet RH after the drier stage was kept around 30% RH, and *κ*_SA,30%_ = 0.75 was used in the calculations. In Hyytiälä the inlet RH was steadily <5% RH, and *κ* of SA was replaced by *κ*_SA,dry_ = 1.18. In both cases the inlet RH was low enough to dry AS and ABS below their efflorescence RH.

To explore the sensitivity of aerosol‐climate interactions to the description of OA water uptake and CCN activation, we set up simulations with two global models, both of which provided input for the Climate Model Intercomparison Project 5 (CMIP5) used by the IPCC in their recent AR5 assessment report [*Stocker et al*., 2013]. NorESM is a fully coupled atmosphere‐ocean general circulation model [*Kirkevåg et al*., 2013; *Bentsen et al*., 2013; *Iversen et al*., 2013]. In this study we used the atmospheric component driven by prescribed, observation based, present‐day sea surface temperatures, similar to the standard model setup used by *Kirkevåg et al.* [2013]. NorESM includes a description of the life cycle of atmospheric aerosol particles, and as a default, the aerosols affect radiation, clouds and climate interactively (“online”) during the simulation. NorESM takes into account climate effects of organic, black carbon, sulfate, dust, and sea salt aerosols. The aerosol description is based on production‐tagged mass concentrations, internally or externally mixed, described explicitly for each of the different modes of the aerosol size distribution (nucleation, Aitken, accumulation, and coarse mode). Aerosol microphysical properties such as effective dry particle size and aerosol optical parameters, including the effect of hygroscopic growth, are estimated by use of interpolations in precalculated look‐up tables which take ambient RH and a range of process‐specific aerosol concentrations from the model as input parameters. Aerosol hygroscopic growth for RH < 100% is estimated using similar look‐up tables. Activation of aerosol particles acting as CCN follows the approach of *Abdul‐Razzak and Ghan* [2000], and the cloud microphysics are simulated with a two‐moment scheme. The aerosol direct and indirect effects on the Earth's radiation budget may be estimated individually via parallel calls to the radiative transfer code. The model sensitivity to hygroscopicity was studied both with or without interactions between aerosols and meteorological conditions (see Table S3 for a list of simulations). In the case without aerosol‐cloud and aerosol‐radiation interactions, the meteorology was identical in all runs (termed also as “offline” as opposed to “online” simulations). The model was set up with a horizontal resolution of 1.9° × 2.5° and 26 levels in the vertical. Besides the simulations with the NorESM, we also used the global aerosol‐chemistry climate model ECHAM‐HAMMOZ (version echam 6.1‐ham2.2‐moz0.9), referred to as ECHAM6‐HAM2 hereon, to study the sensitivity of the present‐day modeled climate to OA hygroscopicity. The aerosol‐cloud‐climate interactions are based on the aerosol module HAM2 [*Zhang et al*., 2012] coupled to the atmospheric general circulation model ECHAM6 [*Stevens et al*., 2013]. HAM2 uses the two‐moment M7 modal aerosol microphysics scheme [*Vignati et al*., 2004] and a two‐moment cloud microphysics scheme that includes prognostic equations for the cloud droplet and ice crystal number concentrations as well as cloud water and cloud ice [*Lohmann and Hoose*, 2009]. The activation of aerosol particles into cloud droplets is parameterized by *Barahona et al.* [2010]. HAM2 calculates the global evolution of five aerosol species: sulfate, organic matter, black carbon, sea salt, and dust. These species are the constituents of both internally and externally mixed aerosol particles whose size distribution is represented by seven unimodal log‐normal distributions. These seven modes describe four size classes (nucleation, Aitken, accumulation, and coarse) and two hygroscopic classes (hydrophobic and hydrophilic). Simulations were performed at 1.9° × 1.9° spectral resolution using 31 vertical levels. Two 5‐year present‐day (years 2006–2010 with 1 month spin‐up) simulations nudged to reanalyzed meteorology from ERA‐Interim [*Dee et al*., 2011] and the corresponding emission inventories as the ones used within NorESM (see Table S4) were conducted: one assuming a *κ*_OA_ of 0.05 and another with a *κ*_OA_ of 0.15.

## 3 Results and Discussion

**Figure 1:**
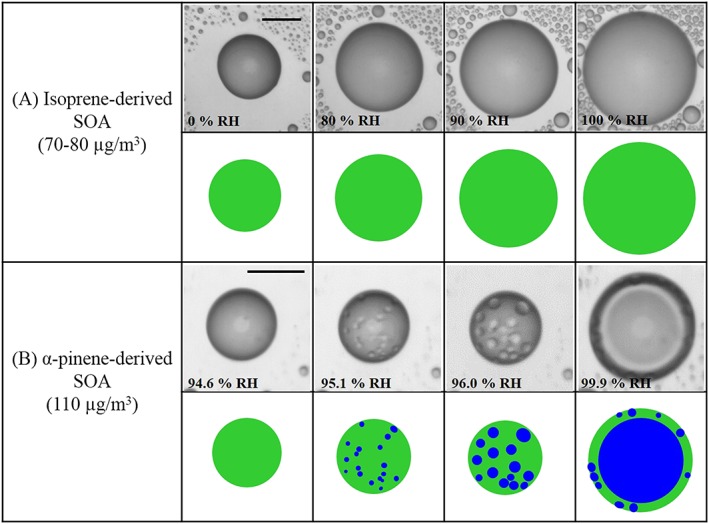
Optical images of micrometer scale SOA particles with increasing relative humidity. (a) Isoprene‐derived SOA for mass concentrations of 70–80 μg m^−3^ and (b) α‐pinene‐derived SOA for a mass concentration of 110 μg m^−3^ [Figure 1a is from the current study while Figure 1b was reproduced from *Renbaum‐Wolff et al*., 2016]. Note that the light gray circles at the center of the particles are due to an optical effect caused by the hemispherical shape of the particles deposited on a substrate. Illustrations are shown below the images for clarity. Green: organic‐rich phase. Blue: water‐rich phase. The scale bar represents 20 μm.

**Figure 2:**
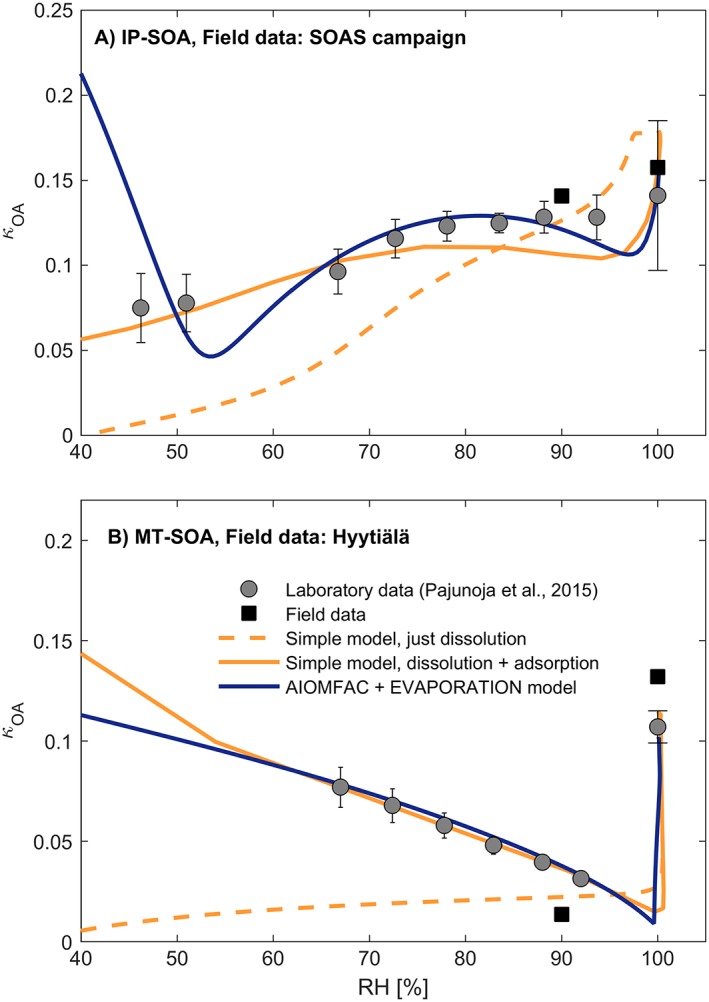
The RH dependencies of the effective hygroscopicity parameter *κ*_OA,eff_ for isoprene‐ and monoterpene‐derived SOA. (a) The hygroscopicity parameter *κ*_OA,eff_ for laboratory‐generated 100 nm particles from IP photooxidation [blue diamonds, O:C = 0.89; *Pajunoja et al*., 2015], organic aerosol sampled at the SOAS site in Alabama (red squares, O:C = 0.63 ± 0.06), and as predicted using two state‐of‐the‐art thermodynamic models (SPARC and AIOMFAC). The SPARC equilibrium calculations are presented for a case accounting for the solubility of the SOA components only (dashed lines) as well as a case including also treatment of adsorptive water uptake and nonideality of the aqueous phase. The AIOMFAC + EVAPORATION calculations account for mixture nonideality, a potential liquid‐liquid phase separation, coupled gas‐particle partitioning of semivolatile organic vapors and water, and a mass‐transfer correction for semisolid organic particles at low RH. (b) Same as Figure 2a but for MT ozonolysis SOA [red diamonds, O:C = 0.56; *Pajunoja et al*., 2015] and organic aerosol sampled at the SMEAR II station in Hyytiälä, Finland (blue squares, O:C = 0.63 ± 0.06). For details of the experiments and the model calculations, see SI.

Our results suggest that the observed differences in *κ*_SOA_ for the biogenic SOA in the subsaturated and supersatured regimes are related to the solubility and phase state. Optical microscopy images of supermicron samples of MT‐ and IP‐derived SOA show that the RH‐dependent phase behavior of these two SOA types is different (Figure 1 and Table S1). For the MT‐SOA a single organic‐rich phase was observed at <95% RH, but at ~95% RH, liquid‐liquid phase separation occurred to form two phases: an organic‐rich and a water‐rich phase (Figure 1b) [*Song et al*., 2012; *Krieger et al*., 2012; *You et al*., 2014; *Renbaum‐Wolff et al.*, 2016; *Petters et al*., 2016]. For IP‐SOA, one single phase was observed over the entire RH range (Figure 1a). This difference in the phase state of IP‐ and MT‐derived SOA particles is also consistent with different RH‐dependent hygroscopic properties of submicron particles from these two precursors (see Figure 2). Laboratory‐generated 100 nm particles derived from the ozonolysis of α‐pinene [*Pajunoja et al*., 2015] with an oxygen‐to‐carbon ratio (O:C) of 0.56 show a marked difference in *κ*_OA_ between subsaturated and supersaturated conditions (denoted here as Δ*κ*_OA_), while corresponding particles of IP‐derived SOA (produced through photooxidation with an O:C of 0.86) show a considerably smaller difference. The laboratory results are consistent with *κ*_OA_ values measured at two field sites (see also SI), namely the SOAS site in Alabama, US (VOC profile dominated by IP albeit with a significant MT contribution) [*Kaiser et al*., 2016] and the SMEAR II station in Hyytiälä, Finland (VOC profile dominated by MT) [*Hakola et al*., 2003; *Raatikainen et al*., 2010; *Finessi et al*., 2012], as well as with values reported previously in the literature for SOA in similar environments [*Pöhlker et al*., 2016]. We can explain this behavior using two independent thermodynamic models with varying levels of complexity (see Figures 2 and S1–S8). Both models indicate that the key process explaining the large Δ*κ*_OA_ in the case of the MT system is the formation of a new aqueous phase in the particles between ~95% and 100% RH, in line with the fraction of OA dissolved. At supersaturation both IP‐ and MT‐derived SOA behave as nearly completely soluble in water in terms of the high *κ*_OA_ values observed, with contributions from potential surface tension reductions as well [*Ruehl et al*., 2016]. The thermodynamics and related phase diagrams can differ between supermicron and submicron sized particles due to the increasing importance of surface/interface energy contributions at smaller sizes, leading to relative preference of the liquid phase at the smaller particle sizes [*Veghte et al*., 2013, 2014; *Werner et al*., 2016; *Altaf et al*., 2016]. Our results, particularly the agreement between the microscopy images and the water uptake of the considerably smaller 100 nm particles, suggest however that phase transition processes govern the water interactions for also the smaller particles in the case of MT‐derived SOA. Hygroscopicity and CCN activity of organic aerosol increases with increasing O:C [*Jimenez et al*., 2009; *Pajunoja et al*., 2015]. As the O:C of the MT‐SOA increases, its hygroscopic behavior becomes increasingly similar to the more oxidized IP‐SOA and Δ*κ*_OA_ decreases [*Pajunoja et al*., 2015].

Bulk‐to‐surface partitioning [*Ruehl et al*., 2016] and gas‐particle partitioning of semivolatile gas‐phase species (co‐condensation) [*Topping et al*., 2011] are two alternative mechanisms that have been proposed to explain the Δ*κ*_OA_ between subsaturated and supersaturated conditions for SOA. However, these mechanisms are not needed to explain the observed Δ*κ*_OA_ between 90% RH and supersaturation when the phase separation effects discussed above are considered. Furthermore, the observed dependencies on the oxidation state, VOC precursor, and RH support the idea of dissolution and/or liquid‐liquid phase separation as the key phenomena to explain Δ*κ*_OA_. As the oxidation state of SOA compounds increases, their water‐solubility also increases due to an increasing number of polar functional groups, resulting in an increase in the associated *κ*_OA_ of the mixture, consistent with our observations. Surface activity of organic species, on the other hand, is expected to increase with increasing hydrophobicity of the molecules, which would result in an opposite dependence of the effective *κ*_OA_ (inferred from laboratory data assuming a constant surface tension of pure water, see SI) on oxidation state than observed at high RH [*Prisle et al*., 2012]. Significant variations in OA mass concentrations due to changes in gas‐particle partitioning with RH, on the other hand, are expected to be more substantial for IP‐SOA with more semivolatile compounds than the MT counterpart (Tables S2 and S3). Constraining the theoretical models of the hygroscopic growth to match experimental data becomes increasingly challenging at RH below 90%, requiring consideration of processes such as adsorptive water uptake, dynamic gas‐particle partitioning of the semivolatile vapors, particle‐phase mass transfer limitations, or considerable nonideality of the liquid phases with decreasing RH.

**Figure 3:**
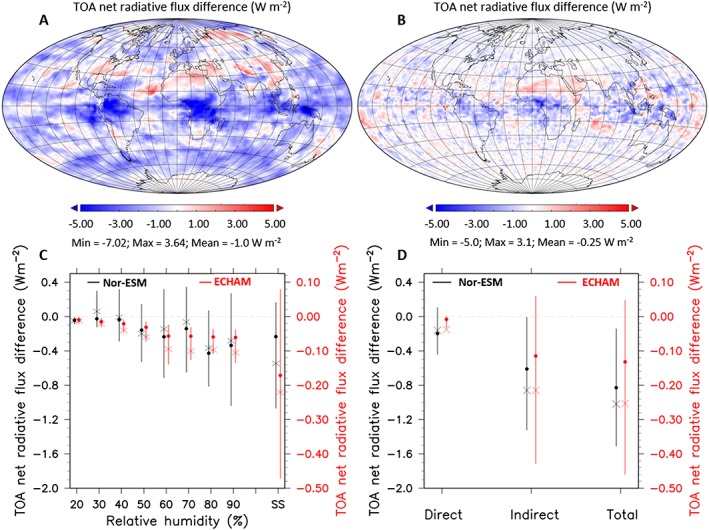
Sensitivities of two CMIP5 climate models to *κ*_OA_. (a) Difference in the top‐of‐the‐atmosphere (TOA) radiative flux in NorESM model simulations of the present‐day atmosphere (22 years simulated) for *κ*_OA_ varying between 0.05 and 0.15 (see SI). (b) Difference in the top‐of‐the‐atmosphere radiative flux in the ECHAM6‐HAM2 model simulations (7 years simulated) of the present‐day atmosphere for *κ*_OA_ varying between 0.05 and 0.15 (see SI). (c) TOA radiative flux difference for *κ*_OA_ varying between 0.05 and 0.15 as a function of RH for NorESM (left axis, black symbols) and ECHAM (right axis, red symbols). Only grid points over land and outside of the polar regions have been considered. (d) The contribution of the direct versus indirect aerosol effects to the model sensitivity for NorESM (left axis, black symbols) and ECHAM (right axis, red symbols). Crosses refer to mean and dots to median values.

Previous studies have suggested that water affinity might play only a minor role in determining the climate impact of OA [*Morales Betancourt and Nenes*, 2014]. The results shown in Figure 3 add a further dimension to the discussion. We used two climate models, namely the atmospheric module of NorESM [*Kirkevåg et al*., 2013] and ECHAM6‐HAM2 [*Zhang et al*., 2012], to study the sensitivity of the Earth's radiative budget to assumptions about organic aerosol hygroscopicity and CCN activity. Both models represent OA with single hygroscopicity, but NorESM has a significantly higher global OA mass concentration (average total OA loading of nearly 4 Tg), while ECHAM6‐HAM2 (average OA loading of about 1 Tg) represents a lower OA loading among the CMIP5 models [*Tsigaridis et al*., 2014]. Both models have an interactive representation of aerosol and cloud processes but differ in the microphysical parameterizations of the aerosol size distribution and cloud hydrometeor number concentrations. NorESM simulates a global average difference of about −1.02 W m^−2^ in aerosol radiative effects between cases with *κ*_OA_ values of 0.15 and 0.05 (Figure 3). This sensitivity to *κ*_OA_ is substantial, considering that the estimated overall climate forcing of anthropogenic aerosol particles during the industrial period is of the order of −1 W m^−2^ [*Stocker et al*., 2013]. ECHAM6‐HAM2, on the other hand, simulates only about one fourth of the NorESM sensitivity to *κ*_OA_ (difference of −0.25 W m^−2^ for *κ*_OA_ values of 0.15 versus 0.05) [*Morales Betancourt and Nenes*, 2014]. The sensitivity in both models is highly regional, being most pronounced over tropical regions (Figures 3a and 3b), and the effects of *κ*_OA_ are largest for RHs over 60%. The indirect effect of aerosol particles on cloud properties dominates the sensitivity as compared to the direct aerosol‐radiation effect (Figures 3c, 3d and S9), but the magnitude of the sensitivity is probably driven by the overall OA loading present in the model.

Given the large uncertainties in the OA loadings in the CMIP models [*Tsigaridis et al*., 2014] with underestimation of OA particularly in urban and marine environments, the global modeling results suggest that constraining the OA water affinity might be more important than previously thought. It is certainly not the only source of uncertainty in climate models, and efforts for improving the spatial model resolution and description of atmospheric dynamics (e.g., updraft velocities and entrainment) need to be pushed forward in parallel with aerosol and cloud microphysics. Besides knowing the source strength of the emissions of various OA types into the atmosphere, improving the OA life cycle requires understanding of the removal mechanisms as well—which, in turn, depend on the OA water affinity. Acknowledging the large variability in the *κ*_OA_ values reported for laboratory and field data on various organic aerosol types [*Lathem et al*., 2013], our results suggest that representing all OA with one constant hygroscopicity parameter can introduce considerable uncertainties to calculations of the climate impacts of OA. Instead, a self‐consistent representation of the climate impacts of OA should rely on an oxidation‐state‐dependent water affinity approach, and ideally this approach would be coupled to both surface phenomena [*Cheng et al*., 2015; *Ruehl et al*., 2016] and a dynamically evolving volatility representation [*Heald et al*., 2010].

## Supporting information



Supporting Information S1
