# Supplementary material for: Microphysical explanation of the RH‐dependent water affinity of biogenic organic aerosol and its importance for climate
Source: Geophys Res Lett. 2017 May 21;44(10):5167–77. doi: 10.1002/2017GL073056 (PMC5518298; doi:10.1002/2017GL073056)
Supplement: Supplementary file 1 — Supporting Information S1 [file GRL-44-5167-s001.pdf]

Geophysical Research Letters

Supporting Information for

Microphysical explanation of the RH-dependent water-affinity of biogenic organic aerosol and its importance for climate

N. Rastak[1], A. Pajunoja[2], J. C. Acosta Navarro[1], J. Ma[3], M. Song[4,5], D. G. Partridge[1], A. Kirkevåg[6], Y. Leong[2,7], W. W. Hu[8,9], N. F. Taylor[10], A. Lambe[11,12], K. Cerully[13], A. Bougiatioti[14,15], P. Liu[16], R. Krejci[1], T. Petäjä[17], C. Percival[18], P. Davidovits[12], D. R. Worsnop[11], A. M. L. Ekman[19], A. Nenes[13-15,20], S. Martin[12], J. L. Jimenez[8,9], D. R. Collins[10], D. O. Topping[18,21], A. K. Bertram[5], A. Zuend[3], A. Virtanen[2], and I. Riipinen[1]*.

[1]Department of Environmental Science and Analytical Chemistry (ACES) and Bolin Centre for Climate research, Stockholm University, Stockholm, 11418, Sweden.
[2]Department of Applied Physics, University of Eastern Finland, Kuopio, 70211, Finland.
[3]Department of Atmospheric and Oceanic Sciences, McGill University, Montreal, QC, H3A 0B9, Canada.
[4]Department of Earth and Environmental Sciences, Chonbuk National University, Jeonju, 54896, Republic of Korea.
[5]Department of Chemistry, University of British Columbia, Vancouver, BC, BC V6T 1Z3, Canada.
[6]Norwegian Meteorological Institute, Oslo, 0313, Norway.
[7]Department of Civil and Environmental Engineering, Rice University, Houston, TX, 77005, USA.
[8]Cooperative Institute for Research in Environmental Sciences, University of Colorado, Boulder, CO, 80309, USA.
[9]Department of Chemistry and Biochemistry, University of Colorado, CO, 80309, USA.
[10]Department of Atmospheric Sciences, Texas A&M University, College Station, TX, 77843, USA.
[11]Aerodyne Research Inc., Billerica, MA, 01821, USA.
[12]Department of Chemistry, Boston College, Chestnut Hill, 02467, MA, USA.
[13]School of Chemical and Biomolecular Engineering, Georgia Institute of Technology, GA, 30332, USA.
[14]School of Earth and Atmospheric Sciences, Georgia Institute of Technology, Atlanta, GA, 30332, USA.
[15]Institute for Environmental Research and Sustainable Development, National Observatory of Athens, Palea Penteli, 15236, Greece
[16]School of Engineering and Applied Sciences, Harvard University, Cambridge, MA, 02138, USA.
[17]Department of Physics, University of Helsinki, Helsinki, 00014, Finland.
[18]School of Earth and Environmental Sciences, University of Manchester, Manchester, M139PL, UK.
[19]Department of Meteorology, Stockholm University, Stockholm, 11418, Sweden.
[20]Institute of Chemical Engineering Sciences, Foundation for Research and Technology-Hellas, Patras, GR-26504, Greece
[21]National Centre for Atmospheric Science (NCAS), University of Manchester, Manchester, M139PL, UK.

Contents of this file

Text S1
Figures S1 to S9
Tables S1 to S4

Introduction

This supporting information contains additional text and figures that provide more detailed insights into the methods and discussion presented in the main text.

Text S1

1. Background: Equilibrium between droplet and water vapor

When an aerosol particle absorbs water and grows in size, the equilibrium size of the formed droplet and its chemical composition are linked to the ambient water vapor saturation ratio (S) through the following equation *[Kohler et al., 1936]*

$$S = \frac{p_{w,eq}}{p_{w,sat}} = a_w \exp\left(\frac{4\sigma v_w}{RT\, D_{p,wet}} \right), \qquad \text{(SI-1)}$$

where $p_{\mathrm{w,eq}}$ (Pa) is the equilibrium vapor pressure of water over the curved surface of an aqueous solution droplet, $p_{\mathrm{w,sat}}$ (Pa) the saturation vapor pressure over a flat pure liquid water surface, σ (N m^{-1}) is the surface tension of the droplet, v_{w} (m^3 mol^{-1}) the molar volume of water proportional to ρ_{w} (kg m^{-3}), the density of water, $D_{\mathrm{p,wet}}$ (m) the diameter of the droplet at equilibrium with the gas phase at saturation ratio S, T (K) the temperature and R (J mol^{-1} K^{-1}) the universal gas constant. The water activity (a_{w}) is defined as the product of the water mole fraction X_{w} and the mole-fraction-based water activity coefficient in the aqueous phase Γ_{w},

$$a_w = X_w \Gamma_w . \qquad \text{(SI-2)}$$

The activity coefficient describes the non-ideal mixing effects between water molecules and the dissolved molecules in the mixture. Setting Γ_w=1 means that the dissolved organic molecules and water are assumed to form an ideal solution (on mole fraction scale). The factor a_{w} in Eq. (SI-1) is related to the Raoult (or solute) effect on the equilibrium vapor pressure of a given compound (here water). The exponential factor in Eq. (SI-1) depends on the droplet size and is known as Kelvin (or curvature) effect.

1.1 Hygroscopicity parameter κ

In many practical applications the water activity (a_{w}) and the difference in the densities and molar masses of water and the dry particle material are approximated using a single hygroscopicity parameter (κ). The simplest form of the definition of κ assumes perfect dissolution and no change in the gas-particle partitioning of particle constituents upon change of RH (i.e., non-volatile solutes), and was introduced by Petters and Kreidenweis (2007):

$$\frac{1}{a_w} = 1 + \kappa \frac{V_s}{V_w} \tag{SI-3}$$

where V_s and V_w are the volumes of the dry material and water, respectively. Assuming that linear molar volume additivity applies, the traditional Köhler equation used for calculating the equilibrium between water vapor and aqueous droplets can be reformulated as the κ-Köhler equation,

$$S = \frac{D_{p,wet}^3 - D_{p,dry}^3}{D_{p,wet}^3 - D_{p,dry}^3(1-\kappa)} \exp\left(\frac{4\sigma M_w}{RT\rho_w D_{p,wet}}\right), \tag{SI-4}$$

where $D_{p,dry}$ is the initial dry particle diameter. This equation can also be formulated as a function of hygroscopic diameter growth factor *HGF*, defined as

$$HGF(S) = \frac{D_{p,wet}(S)}{D_{p,dry}}. \tag{SI-5}$$

As stated above, the simple form of the hygroscopicity parameter κ (Eq. SI-3) does not account for limited water-solubility of the aerosol constituents or their potential re-partitioning between the gas and condensed phases upon changes in the environmental conditions (or inside an aerosol instrument). Furthermore, in the application of Eqs. SI-1 and SI-4 for particles with unknown chemical composition, surface tension of pure water is often assumed. While these uncertain effects can be accounted for in appropriate model calculations (see Sect. 3), they are usually neglected when converting measured hygroscopic growth factors and CCN activities to κ (see Sect. 2).

1.1.1 Effective particle hygroscopicity when accounting for semivolatile compound partitioning

It is worth noting that the particle diameter at humidified conditions is expected to be different from $D_{p,dry}$ for two reasons: (1) water uptake according to the hygroscopicity of the particle's "solute" mixture and (2) change in actual water-free particle mixture composition due to gas-particle partitioning of semivolatile components (which could be a net loss or gain of particulate material). The classical Köhler theory and Eq. (SI-3) of the κ-Köhler theory both ignore the second, partitioning-related point and implicitly assume that all solute material is non-volatile. Accounting for the potential change in solute mixture composition as RH changes, affecting $D_{p,wet}$ and *HGF*, a more general definition of the effective hygroscopicity parameter κ is derived starting from Eq. (SI-4):

$$\frac{1}{a_w} = 1 + \kappa \frac{V_{s,0}}{(V_w + V_s - V_{s,0})}. \tag{SI-6}$$

Here $V_{s,0}$ represents the dry mixture (solute) volume at a reference RH level, typically chosen to be at RH = 0 % (i.e. water-free), while V_s represents the water-free particulate matter volume at the present RH level. Thus, $V_s + V_w = V_p$, with V_p the overall particle volume. Note that Eq. (SI-6) reduces to Eq. (SI-3) when the solute mixture is non-volatile such that $V_s = V_{s,0}$ at all particle sizes and RH levels.

Consistent with Eqs. (SI-4) and (SI-6), the effective particle hygroscopicity is calculated by the following expression:

$$\kappa_{\mathrm{HGF}} = 1 - HGF^3 + \frac{HGF^3 - 1}{S} \exp\left[\frac{4\,\sigma M_w}{RT\rho_w D_{p,wet}}\right]. \tag{SI-7}$$

Equation (SI-7) can be simplified by substituting Eq. (SI-1) for *S*, which leads to cancellation of the Kelvin effect factor exp[…],

$$\kappa_{\mathrm{HGF}} = 1 - HGF^3 + \frac{HGF^3 - 1}{a_w} = (HGF^3 - 1)\left(\frac{1}{a_w} - 1\right). \tag{SI-8}$$

This is meaningful when the actual water activity is known, e.g., in the case of thermodynamic model calculations in which water activity is determined solely by the particle composition (and therefore linked to *HGF*). Equation (SI-8) shows that there is no explicit size dependence of κ_{HGF}, which is expected since κ_{HGF} represents the compositional Raoult' law effect. However, there is an apparent size dependence of κ_{HGF} when plotted versus RH. This is the case because in comparison to the *HGF* of a bulk mixture, the *HGF* of a small droplet (say of 100 nm dry diameter) in equilibrium with its environment is always smaller at a given saturation ratio in the environment (RH or *S*) than that of the bulk mixture – which is what Köhler theory describes. For example, at a fixed temperature, there is a direct relationship between *HGF*, which determines the chemical composition of a particle mixture (including water) and water activity *[Cheng et al., 2015]*. Similarly, for a given mixture composition (expressed by the *HGF*), the water activity is the same for a bulk mixture (large drop) or a small droplet (ignoring a potential composition effect of bulk-to-surface partitioning in the case of surfactants present), yet the saturation ratio in equilibrium with the bulk mixture is equal to its water activity (as seen from Eq. SI-1), while for the small droplet the equilibrium saturation ratio is higher. Hence, a droplet of such a composition and *HGF* could only exist at a higher RH level in the environment than is the case for the corresponding bulk mixture (i.e. a much larger drop).

1.2 FHH adsorption theory

While Eq. (SI-1) describes well the hygroscopic growth and cloud droplet activation of aerosol particles dominated by highly water-soluble compounds, it does not describe the surface interactions through adsorption of water molecules onto insoluble surfaces – although these might become important for the water-uptake of predominantly insoluble particles. For instance, Raymond and Pandis (2002) discovered that organic species with low solubility in water can act as CCN in the atmosphere if they are wettable by water (i.e. their contact angle with water is 0). One possible alternative for explaining the CCN activation behavior of such wettable organic species with low solubility in water is multilayer adsorption theory. The adsorption theory by Frenkel, Halsey and Hill (FHH is one of the most well-known adsorption models and has been widely used *[Frenkel, 1946; Halsey, 1948, 1964; McDonald, 1964; Jiusto and Kockmond, 1968; Hill, 1949; Sorjamaa and Laaksonen, 2007; Kumar et al., 2009, 2011a-b]*. If the surface of the insoluble particle is wettable, the attractive forces between the water molecules and the surface help to form uniform layers of adsorbed water molecules over the particle surface. For particles with negligible solubility, the water activity (a_w) in Eq. (SI-1) can be substituted with the FHH isotherm *[Kumar et al., 2011a]*,

$$a_w = \exp(-A_{FHH}\theta^{-B_{FHH}}) . \qquad \text{(SI-9)}$$

The FHH isotherm is defined by three parameters, namely surface coverage θ (the number of adsorbed water molecules divided by the number of molecules in a monolayer) and two empirical constants (A_{FHH} and B_{FHH}), known as adsorption coefficients. A_{FHH} characterizes interactions of adsorbed molecules with the insoluble aerosol particle surface and adjacent adsorbate molecules (i.e., those in the first monolayer). B_{FHH} characterizes the attraction between the insoluble particle surface and the adsorbate in subsequent layers; the smaller the value of B_{FHH}, the greater the distance over which the attractive forces act.

Combining Eqs. SI-1 and SI-9, the equilibrium saturation ratio for a solution droplet containing an insoluble wettable core is obtained from

$$S = \exp(-A_{FHH}\theta^{-B_{FHH}})\exp\left(\frac{4\sigma M_w}{RT\rho_w D_{p,wet}}\right) . \qquad \text{(SI-10)}$$

1.2.1 Raoult, Kelvin and FHH adsorption effects

The adsorption theory on its own assumes that aerosol particles are insoluble but wettable and can take up water only via surface adsorption. In the case of aerosol particles containing a soluble fraction, however, we need a theory that takes into account the possibility of this soluble fraction being dissolved in the adsorption layer *[Kumar et al., 2009]*. Assuming that the adsorbed water on the surface of the insoluble core is in equilibrium with the

aqueous phase and water vapor, the water activity that accounts for both the Raoult (solute) and adsorption effects is defined as

$$a_w = X_w \Gamma_w \exp(-A_{FHH} \theta^{-B_{FHH}}) . \quad \text{(SI-11)}$$

Therefore, the equilibrium saturation ratio for a solution droplet containing a wettable core surrounded by an aqueous phase (i.e. a mixture of water and the soluble fraction) is obtained from

$$S = X_w \Gamma_w \exp(-A_{FHH} \theta^{-B_{FHH}}) \exp\left(\frac{4\sigma M_w}{RT\rho_w D_{p,wet}} \right) . \quad \text{(SI-12)}$$

Atmospheric aerosol particles contain a large variety of different compounds, and differences in molecular shape and size and interactions between these compounds in aqueous solutions lead to deviations from ideal thermodynamic behavior. As described in the Methods section, we used two approaches with different levels of complexity for describing the non-ideality of the mixtures: a simple model where the non-ideality and dissolution was predicted using a combination of the SPARC prediction tool and the AIOMFAC version available through the UManSysProp website and coupled with FHH theory for adsorption, and a more comprehensive approach where AIOMFAC was used together with EVAPORATION to explore non-ideal mixing and phase transition effects on predicted κ_{HGF} and κ_{CCN}. The latter equilibrium gas-particle partitioning model included the prediction of a potential liquid-liquid phase separation (i.e. a liquid-liquid equilibrium state) and kinetic limitations for water uptake at low RHs *[Paramonov et al., 2013]*. The model was run similar to the case studies by Zuend et al. (2010) to account for the concurrent water uptake and partitioning of semi-volatile organic compounds contributing to the effective hygroscopic growth at a given RH. Predictions from this model and a discussion of the assumptions and results are given in Sect. 3 of this SI.

These two thermodynamic modeling approaches with varying degrees of complexity are schematically summarized in Fig. S1.

2. Supporting experimental methods

2.1 Supplementary information on instrumentation during ambient measurements

The measurements in Alabama at Centreville (32.90289°N, 87.24968°W, 126 m asl) were performed as a part of the Southern Oxidant and Aerosol Study (SOAS) campaign from 10th June to 15th July, while the measurements in Hyytiälä SMEAR II station (61.84524°N, 24.28883°E, 181 m asl) were conducted from 9th May to 3rd June. Both sites are rural sites in a forest environment, with minor influence of local anthropogenic emissions on total

aerosol concentrations sampled during the campaigns. Particle water uptake properties and chemical composition were investigated with equal methods in both sites; CCNc was used to study particle activation to cloud droplets. HTDMA was used to measure hygroscopic growth at sub-saturated conditions and HR-ToF-AMS was used to derive chemical composition of submicron particulate matter (PM_1). A set of other measurements were made at the same time, and are shown elsewhere, *[Paramonov et al., 2013; Hu et al., 2015; Cerully et al., 2015; Xu et al., 2015; Guo et al., 2015]*.

2.2 Meteorology and aerosol characterization

Both measurement sites comprise weather stations with continuous measurements of years prior to and after the campaigns. Evolution of aerosol populations in the ambient air was monitored with DMPS at both sites, and the averaged number size distributions are shown in Figs. S2-A and S3-A.

2.3 CCNc

Size-resolved CCN measurements were done with Droplet Measurement Technologies continuous-flow streamwise thermal gradient CCNc *[Roberts and Nenes, 2005; Paramonov et al., 2013; Cerully et al., 2015]*. Dried and size-selected ambient particles were pulled through a fixed supersaturation (in this study: 0.2%) and the activated particle number concentration was compared to the initial number concentration of dried particles. Based on κ-theory *[Petters and Kreidenweis, 2007]*, CCNc results can be transformed to hygroscopicity, κ, which then can be compared to sub-saturation water uptake measurements, such as with HTDMA. The CCNc setup and measurement procedure conducted in Centreville and in Hyytiälä are explained in more detail elsewhere *[Paramonov et al., 2013; Pajunoja et al., 2015]*, respectively.

2.3 HTDMA

HTDMA was used to measure hygroscopic growth of aerosol particles at sub-saturated conditions *[Brechtel and Kreidenweis, 2000; Hong et al., 2014; Pajunoja et al., 2015]*. Sample air was dried below 30 RH% and then charged with a bipolar charger. Then a monodisperse aerosol distribution was selected with a DMA (Vienna type, custom made). The selected monodisperse particle population was passed through another DMA in which the sheath air was humidified to a fixed RH (90 ± 2%). The second DMA was used in scanning mode paired with a CPC to derive the number size distribution of the wetted aerosol population. The hygroscopic growth factors (HGF), which are based on the ratio of the medians of the dry and wet particle number distributions, were calculated at the measured RH and then transformed to the respective hygroscopicities, κ. The details of the HTDMA measurements made in Alabama and Hyytiälä can be found elsewhere *[Cerully et al., 2015; Pajunoja et al., 2015]*.

2.5 AMS

Particle mass spectra were measured with an Aerodyne High Resolution Time of Flight Aerosol Mass Spectrometer (HR-ToF-AMS, hereafter: AMS) *[DeCarlo et al., 2006]* in Alabama and with an Aerodyne compact ToF-AMS (hereafter: AMS) in Hyytiälä. Average elemental oxygen to carbon ratios (O:C) of the particles were calculated according to Aiken et al. (2008) and Chen et al. (2011), but also converted to follow the revised method described by Canagaratna et al. (2015).

3. Interpretation of the laboratory and field observations

The time series of ambient measurements are shown in Figs. S2 and S3. While nucleation events appear daily in Hyytiälä (see Fig. S2A), the number of events remains low at the Alabama site (see Fig. S3A). The actual PM_1 concentration stays in a similar range at both sites (Figs. S2B and S3B, black line) and the organics are contributing the most to PM_1 (green line in Figs. S2B and S3B). Hygroscopicities (as κ values) at both sites have clear diurnal variation peaking during daytime (Figs. S2C and S3C). Similar behavior can be seen for the average oxidation level (by means of O:C) of the particles (grey dots in Figs. S2B and S3B). Nevertheless, hygroscopicity of SOA particles is different between the two sites; while κ_{HGF} (here at 90 % RH) and κ_{CCN} (here 0.2 % SS) in Alabama follow the same line (Fig. S3C), κ_{HGF} is clearly less than κ_{CCN} in Hyytiälä (Fig. S2C). Previous studies have shown that IP is a dominant gas-phase VOC in Alabama and that a major fraction of the SOA consists of IP-derived SOA and other highly oxidized species *[Cerully et al., 2015; Xu et al., 2015]* while monoterpene (MT) SOA typically comprises a major fraction of the atmospheric aerosol in Hyytiälä *[Raatikainen et al., 2010; Finessi et al., 2012]*. The experimental laboratory data shows a significant discrepancy between κ_{HGF} and κ_{CCN} for MT-SOA, which is in line with the ambient data from Hyytiälä, while the discrepancy is negligible for IP-SOA *[Pajunoja et al., 2015]*, in line with the ambient data from Alabama.

3.1 Calculations with the simple dissolution + adsorption model

To investigate the hygroscopic growth and cloud droplet activation behavior of multicomponent organic particles, we used the water-solubility distributions of MT and IP SOA *[Hilal et al., 1995]* (Fig. S4). The organic mixture composition (see Tables S2 and S3) and dry particle size (100 nm) were used as model inputs. The wet particle size and final equilibrium composition of organics are determined by considering the equilibrium between the water vapor and the aqueous phase on one hand and the aqueous phase and the potential insoluble organic phase on the other hand. The wet particle size and composition is then linked to the ambient water vapor saturation ratio S (or RH) using Eq. SI-1, assuming either an ideal aqueous solution or accounting for the aqueous phase non-ideality by using the AIOMFAC model. The SOA surrogate molecules[28], predicted by the Master Chemical Mechanism (MCM; http://mcm.leeds.ac.uk/MCM), were used for

predicting solubility distributions of isoprene and monoterpene SOA by the SPARC model *[Hilal et al., 1995; Riipinen et al., 2015]*. In our calculations, the mole-weighted average molar masses of 150 g mol^{-1} and 195 g mol^{-1} and the average mass densities of 1480 kg m^{-3} and 1230 kg m^{-3} were used for IP and MT, respectively.

To take into account the role of adsorption, the surface coverage θ was calculated using the dissolved organic fraction determined by the dissolution model described above and the adsorption coefficients (A_{FHH}, B_{FHH}) fits to the laboratory data. In the case of a large fraction of soluble material the adsorption term must approach unity, leaving the Raoult term (dissolution) to take over in controlling the water uptake. In previous studies conducted for fully insoluble and relatively large particles *[Sorjamaa and Laaksonen, 2007; Kumar et al., 2009, 2011a,b]* the surface coverage θ was defined as

$$\theta = \frac{r_{p,wet} - r_{p,dry}}{2r_w} \qquad \text{(SI-13)}$$

where $r_{p,wet}$ is the droplet radius, $r_{p,dry}$ is the dry initial radius and $r_w = 0.14$ nm is the equivalent radius of a water molecule adsorbed on the surface. Accounting for a potential soluble fraction requires reformulating θ to express a modified, effective surface coverage. The number of monolayers equals the total number of water molecules in the droplet divided by the number of water molecules in each layer. The reformulated θ is then defined as

$$\theta = \frac{r_{p,wet}^{3} - r_{core}^{3}}{4r_w r_{core}^{2}} \qquad \text{(SI-14)}$$

where r_{core} is the radius of the potential insoluble core surrounded by the aqueous phase. This formulation can be applied to particles regardless of their soluble fractions. It is notable, however, that this formulation assumes spherical droplets with an insoluble core in the middle, thus being a coarse approximation for situations where the insoluble material populates the droplet surface instead *[Song et al., 2012; Krieger et al., 2012; Renbaum-Wolff et a., 2016]*. This issue is further addressed in the more complex model calculations accounting for potential liquid-liquid phase separation, vapor re-partitioning, and surface tension reduction effects in Sect. 3.2.

If we assume that at a given RH a certain concentric layer in a spherical particle is dissolved in water with thickness r_{layer} encapsulating an insoluble spherical core of radius r_{core}, we have $r_{core} = r_{p,dry} - r_{layer}$. Assuming that the insoluble and aqueous phases can be described as a quasi-binary system, we can write the amounts of substances of water and solute as

$$n_w = \frac{4}{3}\pi(\mathrm{HGF}^3 - 1)r_{p,dry}^3 \frac{\rho_w}{M_w}\text{, and } n_s = \sum_i n_i = \frac{4}{3}\pi\left(r_{p,dry}^3 - r_{core}^3\right)\frac{\rho_{OA}}{M_{OA}}, \qquad \text{(SI-15)}$$

where the average density and molar mass for the organic mixtures are used as representative for the solute. Using n_w and n_s from Eq. SI-12 to express X_w, and θ from Eq. SI-11, we can rewrite Eq. SI-9 as

$$S = \frac{(\mathrm{HGF}^3-1)r_{p,dry}^3\frac{\rho_w}{M_w}+\left(r_{p,dry}^3-r_{core}^3\right)\frac{\rho_{OA}}{M_{OA}}}{(\mathrm{HGF}^3-1)r_{p,dry}^3\frac{\rho_w}{M_w}} \cdot \exp\left\{-A_{FHH}\left[\frac{\left(\mathrm{HGF}\cdot r_{p,dry}\right)^3-r_{core}^3}{4r_w r_{core}^2}\right]^{-B_{FHH}}\right\}\cdot$$
$$\exp\left(\frac{2\sigma M_w}{\mathrm{HGF}\cdot r_{p,dry}\rho_w RT}\right), \qquad \text{(SI-16)}$$

if we assume that Γ_w=1. The adsorption coefficients A_{FHH} and B_{FHH} were fitted to Eq. SI-16 using laboratory data for *HGF* and *S [Pajunoja et al., 2015]*. To test the importance of potential aqueous phase non-ideality, the online property calculator UManSysProp (Sect. 3) was used to calculate the water activity coefficients (Γ_w) in the aqueous phase, assuming the aqueous phase to contain a dissolved organic fraction (determined by the bulk solubilities) and water. The relative amounts of these were accounted for by varying the mole fraction of water. The insoluble volume was added to the calculation afterwards to reproduce the droplet size (i.e. Kelvin effect).

Figures S5 and S6 show the hygroscopicity data accounting for dissolution and adsorption, calculated using the modeling approach described in Sect. 4.1. Adding adsorption to the solubility model can reproduce the experimental data both for IP and MT at RH < 90%, but the determined adsorption coefficients (A_{FHH}, B_{FHH}) are unrealistically high. A_{FHH} is expected to range from 0.1 to 3.0, while B_{FHH} ranges from 0.5 to 3.0 *[Sorjamaa and Laaksonen, 2007]*. A_{FHH} = 88.1 and B_{FHH} = 2.51 were reported for α-pinene with O:C = 0.45 *[Pajunoja et al., 2015]*. Comparing the case of ideal mixing in the aqueous phase (Fig. S5A and B) with the case including non-ideal mixing using AIOMFAC-predicted activity coefficients in (Fig. S5C and D), shows that accounting for the non-ideality results in a better agreement with the laboratory measurements for the IP-derived SOA. The same comparison for MT-derived particles (Figs. S6A and B vs. Figs. S6C and D) shows that non-ideality in the aqueous phase (with a portion of the soluble organic compounds dissolved) can explain a some of the decreasing trend in κ values with increasing RH, but it is not the only explanation for the observed water-uptake behavior at low RHs.

Figures S5D and S6D show the modelled data when accounting for all processes so far discussed together (i.e. dissolution, adsorption and non-ideality). The results show that by considering all these processes we can predict the water uptake behavior of IP and MT at both the intermediate and high RH ranges to very good agreement. Adsorption coefficients (A_{FHH}, B_{FHH}) in those combined model predictions have more reasonable values, except A_{FHH} for MT, which has a high value compared to previous studies, although the value is

lower than in the case considering only dissolution and adsorption (i.e. Fig. S6B) *[Pajunoja et al., 2015]*. However, it should be noted that the adsorption terms are obtained by a fit of the adjustable adsorption coefficients to the experimental data, and thus, do not rule out other potential explanations.

3.2 AIOMFAC-EVAPORATION calculations accounting for phase separation and gas-particle partitioning

The different modeling approaches discussed above suggest that alternative explanations for the measured hygroscopic growth may exist. For instance, with a somewhat different modeling approach Renbaum-Wolff et al. (2016) can reproduce the water-uptake behavior of MT-SOA with a very good resemblance to our experimental data.

To explore the extent to which a state-of-the-art thermodynamic equilibrium model for gas-particle partitioning and liquid-liquid phase separation can explain the measured variations of κ_{HGF} with RH and the discrepancy to κ_{CCN}, we conducted computations with the AIOMFAC-based model framework *[Compernolle et al., 2011; Zuend and Seinfeld, 2012]*. The gas-particle partitioning model was operated on the assumption that the SOA mixtures are in a liquid (potentially viscous) state and may therefore absorb (rather than adsorb) an amount of water depending on the level of RH. In addition, the model includes the algorithm developed by Zuend and Seinfeld (2013) for the prediction of a potential liquid-liquid phase separation as a function of overall liquid mixture composition.

A liquid-liquid phase separation (LLPS), i.e. a miscibility gap, may occur in mixtures of rather dissimilar compounds (e.g. in terms of polarity, degree of oxidation, or density) and can be predicted by appropriate thermodynamic models as a result of non-ideal molecular interactions *[Compernolle et al., 2011; Zuend and Seinfeld, 2013]*. In the context of limited solubility, one can consider a LLPS to be an example of limited solubility of organic mixture components in one of the liquid phases, e.g. limited solubility of nonpolar compounds in the water-rich aqueous phase in comparison to the preferred, low-polarity organic-rich phase. Hence, it can be expected that LLPS could explain some of the observed features for the MT SOA system. Isoprene SOA contains compounds of higher O:C ratios, such that a LLPS is not expected for that system. Extensive experimental studies have shown that mixtures with an average O:C ratio above 0.8 are unlikely to undergo LLPS at atmospherically relevant RH – even in the presence of dissolved electrolytes that induce a salting-out effect on species of lower polarity *[Bertram et al., 2011; You et al., 2014]*.

The pure component liquid state vapor pressures for the organic SOA components were computed for a temperature of 298.15 K using the EVAPORATION model. The calculated vapor pressures of the organic components range from low- to semi- and intermediate volatilities, which means that for some compounds a significant fraction of the material

will be present in the gas phase at equilibrium (e.g. when the gas phase volume is of 1 m^3) or may evaporate during the experiment. This is particularly the case for several of the components of the IP SOA system. Tables S2 and S3 list the predicted particle-phase mass fractions for 3.6 µg m^{-3} of SOA at 40% RH *[Chen et al., 2011]*. A tetrol-dimer compound, typically found in isoprene SOA under low-NO_x conditions *[Surratt et al., 2010]*, was added to the IP SOA system to produce a SOA mixture with an average O:C ratio that matches the one of the laboratory experiments (otherwise, the O:C ratio of the MCM-predicted IP mixture is greater than 1). First, the molar amount of each SOA compound was calculated based on those mass fraction values and scaled to reach a total SOA mass concentration in agreement with the measurement conditions. The gas-particle partitioning model was then run at 40% RH to estimate the molar amounts of the different organics that would be present in the gas phase at equilibrium with such a particle. This also resulted in a prediction for the total molar amounts of all species in a unit volume of air. Second, in order to mimic the conditions of the HTDMA measurements, this initial total molar amounts were further adjusted to account for evaporative losses of the gas phase during the drying stage of the aerosol prior to entering the HTDMA. In other words, a large fraction of the rather volatile components was removed during the drying process alongside with water vapor – and this needs to be considered in the model to establish conditions similar to the experiments. We therefore run the gas-particle partitioning model to calculate the equilibrium gas phase amounts at ~1% RH based on the initial molar input. The gas phase amounts were then removed and the remaining particle phase molar amounts were taken as the new total molar amounts. This process was repeated once, to remove most of the intermediate volatility compounds, consistent with evaporative loss of semi-volatile material during the drying stage of HTDMA experiments *[Zhang et al., 2015]*. The total (gas + particle phase) molar amounts determined by this procedure are listed in Tables S1 and S2 for the IP and MT systems, respectively. We note that without such a modification of the initial total molar SOA composition, the final model predictions would deviate considerably from the measured hygroscopic growth data in the case of the IP system. Our model test-runs revealed that this was a crucial step in the preparation of the SOA amounts to be used for hygroscopicity calculations with the AIOMFAC-EVAPORATION model (i.e. mimicking the processes occurring in a HTDMA experiment).

The AIOMFAC-based gas-particle partitioning model was run for several contrasting model scenarios, with the goal to account for different possibilities of gas-particle equilibration of the organic SOA compounds during the residence of the particles in the HTDMA instrument. The emphasis was on the goal to reproduce the observed hygroscopicity behavior of the SOA systems by accounting for the experimental conditions and additional information about the aerosols phase state (liquid, semi-solid/viscous, or solid) at different RH levels by making use of the measured particle bounced fraction (BF) *[Pajunoja et al., 2015]*. We note that for substantially different experimental/environmental conditions the model predictions may deviate from the ones

presented in this study. Two relevant calculation scenarios are discussed in the following, with Scenario 2 assumed to match the experimental conditions best.

Scenario 1: No organic gas-particle partitioning. It is assumed that the SOA compounds are not equilibrating/evaporating rapidly enough to changes in the (depleted) gas phase composition during the short residence time in the HTDMA instrument. Therefore, the organic mixture components are treated as non-volatile, while water is considered to reach equilibrium with the set RH level quickly during the hygroscopicity measurement.

Scenario 2: Bounced-fraction-adjusted gas-particle partitioning. It is assumed that aside from water also SOA components partition between the gas phase and the particles in the humidified section of the HTDMA, but the equilibration time scale is impacted by the viscosity of the mixture. The idea is that a viscous particle mixture at lower RH levels will lead to slow evaporation/condensation rates, especially impacting the equilibration of semi-volatile organics on the order of ~8 s residence time in the instrument. Good model-measurement agreement was found when the equilibrium vapor pressures of individual SOA components were scaled by a 4-parameter sigmoidal function that is based on measured particle bounced fraction. The following function was used to calculate the fraction of non-bouncing (lower viscosity) particles f_{BF} (= 1 – BF) as a function of (fractional) RH:

$$f_{\mathrm{BF}} = a + \frac{1-a}{\left[1+\left(\frac{\mathrm{RH}}{b}\right)^{c}\right]^{d}} . \tag{SI-17}$$

Here, a, b, c, and d are adjustable function parameters that were fitted such that $(1 - f_{\mathrm{BF}})$ matches to good approximation the measured BF data. The determined parameters are: a = 0.18, b = 0.52, c = -40, d = 0.18 for IP-SOA and a = 0.15, b = 0.87, c = -50, d = 0.20 for MT-SOA. We note that for the IP system, the fit value of parameter b was 0.60, but this value was further adjusted to 0.52 in order to achieve better agreement of the model predictions with measured κ_{HGF} data. This correction for parameter b shifts the effective onset of a viscosity effect on the equilibration time from 60 % RH to 52 % RH. This is justified given that the actual evaporative loss in the instrument depends both on the evaporation rate and the time spent for evaporation/equilibration in the instrument. The application of function f_{BF} leads to an appropriate (relative) scaling of the SOA vapor pressures (change in effective evaporation rate with changing RH) in agreement with a mixture viscosity effect that depends predominantly on the particle water content, which is controlled by RH. We also note that this BF-based adjustment to the SOA vapor pressures is mostly relevant for the IP system, for which the predicted hygroscopic growth and κ_{HGF} values are quite sensitive to the effective volatility distribution. In contrast, the MT system consists already of sufficiently low-volatility compounds such that the f_{BF}-adjustments lead to negligible changes in model predictions compared to Scenario 1.

Resulting model predictions for the hygroscopic growth properties of the IP-SOA system are shown in Fig. S7. Figure S8 shows the corresponding model predictions for the MT-SOA system. The model predictions were done for a bulk mixture (for which the Kelvin effect is absent) as well as for particles of 100 nm dry diameter with constant droplet surface tensions of either 72 mN m^{-1} (the value for pure water at 298 K) or an assumed value of 50 mN m^{-1} (a value indicative of surface tension reduction by organic compounds at the air-droplet interface prior to droplet activation). We note that assuming a fixed, reduced particle surface tension is a rough estimate, serving the purpose of exploring the effects of such a lower surface tension on the value range of the effective hygroscopicity parameter. As discussed by Ruehl et al. (2016), the actual surface tension of growing aqueous organic particles will vary, depending on the composition and phases present, and will approach the surface tension value for pure water as the particle grows toward and/or beyond its cloud droplet activation size. Panel B of Figs. S7 and S8 shows the computed κ_{CCN} with κ_{HGF} (from Eq. SI-8) as a function of saturation ratio. The indicated κ_{CCN} is simply the predicted κ_{HGF} value at the highest equilibrium supersaturation value predicted - corresponding to the global maximum in a Köhler curve. The error bars denote the numerical precision with which the maximum is resolved.

The thermodynamic model does not predict a LLPS for the IP-SOA system at any RH, in agreement with the expectation for an average organic O:C of ~0.86. However, the model-measurement comparison for *HGF* and κ_{HGF} show that this system is very sensitive with respect to the degree of evaporative re-partitioning of SOA components at different RH levels. The measured *HGF* data can only be matched reasonably well by the model when the IP-SOA is treated as semivolatile and this is key to achieve good agreement with the measured RH-dependent variations in κ_{HGF}. The model calculation with BF-adjustment (Scenario 2) suggests that at low RH, the evaporative loss (over a short time of a few seconds) is limited by a relatively viscous physical state, while the evaporative loss increases at RH levels above 52 %. It is interesting to note that at high RH (> 90 %), the predicted evaporative loss is small again (i.e. most SOA partitions increasingly to the particle phase at higher RH) due to sufficiently abundant amounts of particle-bound water, shifting the equilibrium toward the liquid phase and leading to an increase in the computed effective κ_{HGF}.

In the case of the monoterpene SOA system, the AIOMFAC-based prediction for *HGF* and κ were slightly higher than measured when the MCM components and molar amounts form Chen et al. (2011) were used directly. We concluded that it is likely that some important extremely low volatility compounds (ELVOCs) are missing in this case, e.g., dimers formed from monomeric precursors (with the monomers and their molar yields predicted by MCM). Dimers are commonly observed in MT ozonolysis and formed rapidly at the beginning of MT oxidation experiments *[Zhang et al., 2015]*. ELVOCs could also account for ~15 % to 40 % of the total organic mass in the particle phase *[Petters et al., 2016]*. In

a previous modeling study *[Zuend and Seinfeld, 2013]*, one "Ester dimer" (dimer from ester formation of precursor monomers) and one "Aldol dimer" (dimer via aldol condensation) were added to an MT-SOA system to better match experimental SOA yields by the gas-particle partitioning model predictions. The experiments by Pajunoja et al. (2015), data from which are used in this study, may also have led to dimer formation. To consider such dimers in this MT model system, we introduced one "Ester dimer" compound formed from the precursor monomers pinic acid and hydroxypinonic acid. As shown in Table S2, 99 % (by moles) of hydroxypinonic acid (HOPINONIC) was used to constrain the amount of Ester dimer (as HOPINONIC is the limiting monomer here). Figure S8 shows the model predictions for this MT-SOA system composition. Good agreement between model predictions and the measured hygroscopic growth factors is achieved. The Ester dimer constitutes about 20 % of the total organic mass in the particles, which is within the mass fraction range of ELVOC amounts reported by Zhang et al. (2015)[44] for SOA formed by α-pinene + ozone reactions. The Ester dimer is only moderately polar (O:C of 0.368), which slightly decreases the average O:C value predicted for the organic mixture to ~0.52 and is also partially responsible for the occurrence of a liquid-liquid phase separation. A LLPS is predicted in the high-RH range between 98.40 % and 99.986 % RH in the case of the bulk solution calculation, denoted by the vertical yellow lines in Fig. S8, and in conceptual agreement with the predictions of the simple dissolution + adsorption model (see previous section). For particles of D_0 = 100 nm and σ = 72 mN m^{-1} the LLPS range is predicted to cover the range from 100.19 % to 100.41 % RH. The phase transition from a single, organic-rich phase to a LLPS state is accompanied by a sharp increase in particle water content, as is clearly seen in both the steep increase in HGF at those high RH levels and in the change in mass fraction of water in the overall particle (Fig. S8, panels A, C). This sharp transition in water content is due to LLPS leading to the formation of an aqueous phase with dissolved SOA products of higher water-solubility, while less polar organics remain in the organic-rich phase. Once the water content is high enough (above a_w = RH = 99.986 % in the bulk mixture) all SOA compounds are dissolved in a single aqueous phase, which marks the upper end of the LLPS range predicted for the MT system here. Similar effects of high-RH LLPS on particle growth were found for other SOA mixtures in two recent studies *[Renbaum-Wolff et al., 2016; Petters et al., 2016]*. In contrast to the IP-SOA system, the MT-SOA mixture is dominated by lower volatility compounds and the equilibration of semi-volatile species with the gas phase does only lead to minor changes in the particle composition and hygroscopic properties.

A reduced surface tension due to enhanced partitioning of lower-polarity organic compounds to the surface and/or the persistence of an organic phase at the particle surface into the supersaturated range, may lead to a significant impact on the particle diameter prior to and at CCN activation *[Renbaum-Wolff et al., 2016]*. Figure S8B indicates that the assumption of a reduced droplet surface tension has an influence on the predicted κ_{CCN} in

this case), because κ_{CCN} calculated for a surface tension of 50 mN m^{-1} is in slightly better agreement with the experimental value than the one calculated for 72 mN m^{-1}. However, in terms of impact on κ_{HGF} and κ_{CCN}, of primary interest in this study, the two systems show that the variation of the hygroscopicity parameter with different assumptions about the particle surface tension is relatively minor when contrasted by the much larger effect of κ_{HGF} evaluated at different sub-saturated RH levels in comparison to κ_{CCN}. In conclusion, both approaches using a predicted solubility distribution and adsorption model, as well as the AIOMFAC-based thermodynamic phase behavior prediction with potential LLPS, offer explanations of the experimental findings – and both approaches reproduce the limited water uptake of MT-SOA at RH < 98 %. The thermodynamic gas-particle partitioning calculations reveal that both LLPS and gas-particle partitioning of semivolatile species may have a significant impact on measured (and ambient) κ_{HGF} in the subsaturated RH regime as well as on the aerosol water uptake at supersaturated conditions and the related κ_{CCN} parameter. These calculations offer plausible explanations for the hygroscopicity and CCN activation measurements by Pajunoja et al. (2015).

4. Supplementary results from NorESM simulations

We have investigated the sensitivity of climate predictions to hygroscopicity of organics with NorESM by using different κ values for tropospheric organic aerosols (see Table S4). Here we present the difference between two simulations, one with κ_{OA} = 0.15 (high case) and the other one with κ_{OA} = 0.05 (low case) as an example. Figures S9A and S9B show the simulated direct and indirect effects of aerosols (natural and anthropogenic) in terms of radiative forcing at the top of the atmosphere (Wm^{-2}) for the difference between these two cases (high minus low). The model was here run in offline mode, in which the feedback of aerosol property changes on the atmospheric dynamics is switched off. The largest impact of the changes in organic aerosol hygroscopicity is seen over the continental tropical regions, where a higher loading of organic particles and water vapor is typically found simultaneously. The sensitivity of the indirect aerosol effect to κ_{OA} is higher than for the direct effect, with a simulated global mean of -0.11 Wm^{-2} for the direct effect and -1.12 Wm^{-2} for the indirect effect.

The online mode calculations (with aerosol feedback on the atmospheric dynamics) do not provide direct and indirect aerosol radiative forcing (Wm^{-2}) as model output. Therefore, we use three other output parameters that are comparable with radiative forcing in the case of the online mode simulations. Figure S9C shows the FSNTC (Clear Sky Net Solar Flux at Top of the Atmosphere) with a global mean value of -0.26 Wm^{-2}. This parameter is comparable with the direct radiative forcing (DRF) reported in the case of our offline mode calculation. Figure S9D shows the SWCF (Short Wave Cloud Forcing) with a global mean value of -0.86 Wm^{-2}, comparable with the indirect radiative forcing (InRF). Figure S9E shows the FSNT (Net solar flux at Top of the Atmosphere) with a global mean value of -

1.20 Wm^{-2}, comparable with the total direct and indirect radiative forcing prediction. The variations in FSNTC are lower than for SWCF, consistent with the offline calculations yielding a larger indirect than direct effect. The regional changes accompanying online mode calculations are more complex than in the offline case since the aerosols are allowed to affect the atmospheric dynamics, leading to an interplay of feedback mechanisms. The overall magnitude of the sensitivity to κ_{OA}, however, is similar in both calculations.

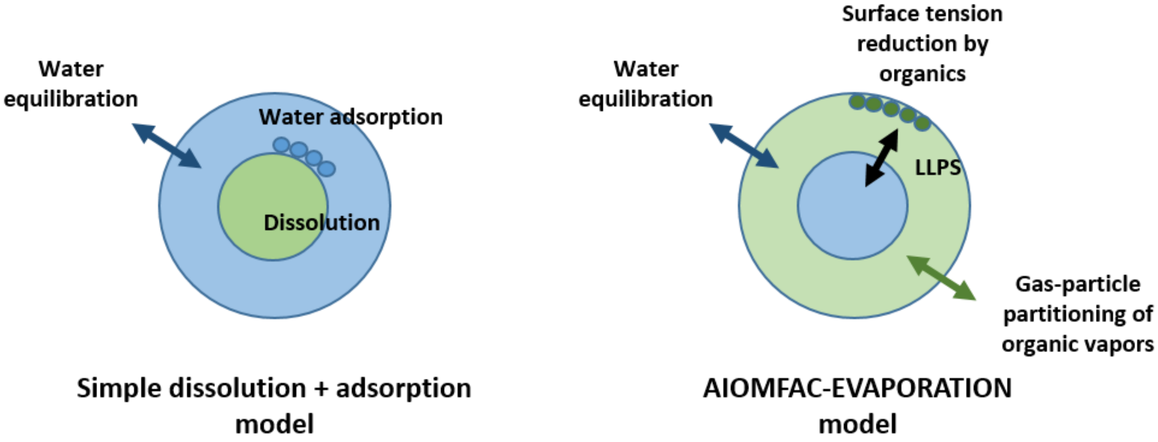

Figure S1. Schematic representation of the two thermodynamic modeling approaches used to calculate the hygroscopic water uptake and CCN activity of the IP- and MT-derived SOA. Both models used AIOMFAC for describing the aqueous phase water activity. The simple dissolution model used SPARC for calculating the organic component solubilities, while the AIOMFAC-EVAPORATION equilibrium model includes an algorithm for the prediction of a liquid-liquid phase separation (LLPS), potentially present in a system (such as aqueous MT-SOA). Furthermore, the simple model assumed surface tension of water, and potential water-uptake by adsorption at low RHs. The AIOMFAC-EVAPORATION approach, on the other hand, was also used to explore the effect of surface tension reduction by organics, as well as the coupled gas-particle partitioning of semi-volatile organic molecules and water upon RH changes (with potential kinetic limitations during equilibration related to the organic phase state) in the HTDMA setup.

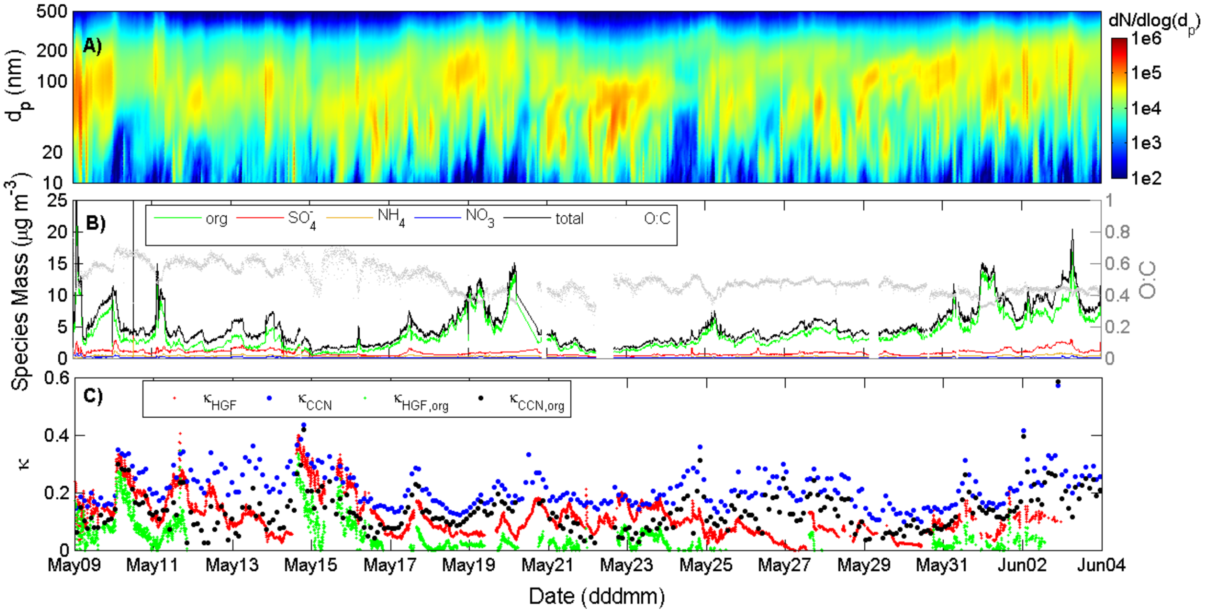

Figure S2. Time series of ambient data measured at the SMEAR II station, Hyytiälä on 9th May to June 4th in 2013. A) Number size distribution data of ambient particles between 10-500 nm, shown as dN/dlog(d_p), B) composition of PM_1, mass species and oxygen-to-carbon ratio (O:C, grey dots) derived from AMS results, and C) hygroscopicity values κ calculated from CCNc (blue and black dots) and HTDMA (red and green dots) measurements. Hygroscopicities of organic fractions (black dots for CCNc and green dots for HTDMA) are based on the method presented by Nenes et al. (1998).

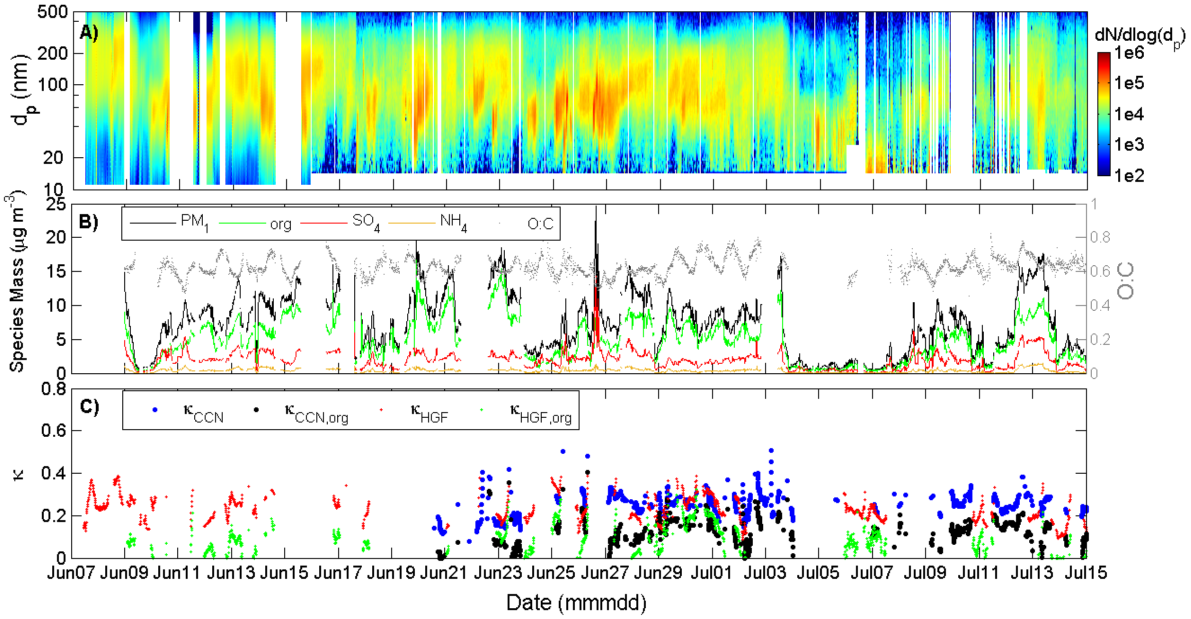

Figure S3. Time series of ambient data measured at Centreville, Alabama on 7th June to July 15th in 2013. In panel A) number size distribution data of ambient particles between 10-500 nm, dN/dlogDp, B) composition of PM_1, mass species and degree of oxidation (O:C, grey dots) derived from AMS results, and C) hygroscopicity κ values calculated from CCNc (blue and black dots) and HTDMA (red and green dots) measurements. Hygroscopicities of organic fractions (black dots for CCNc and green dots for HTDMA) are based on the method presented by Nenes et al. (1998).

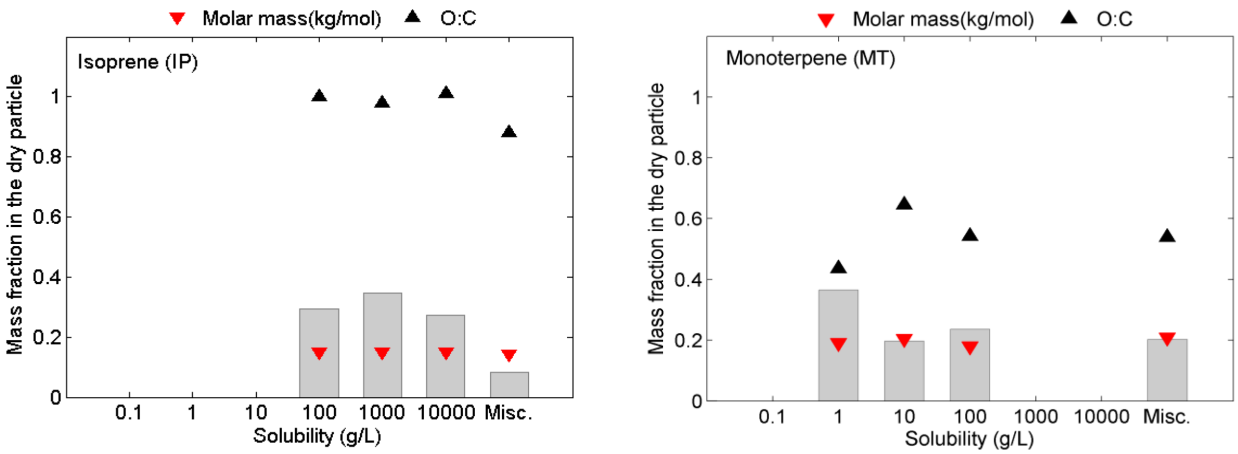

Figure S4. A) The solubility distribution assumed for isoprene (IP). The solubility value for Misc (miscible) bin was 10^5 gL^{-1}. IP with a high oxygen-to-carbon ratio (O:C = 1.07) has high water solubility, ranging from 100 g L^{-1} to fully soluble. B) The solubility distribution assumed for monoterpene (MT). The solubility value for Misc (miscible) bin was 10^5 gL^{-1}. MT with (O:C = 0.54) is less soluble in water than IP with values mainly between 0.1 and 100 gL^{-1} and about 20% of mass in the Misc bin.

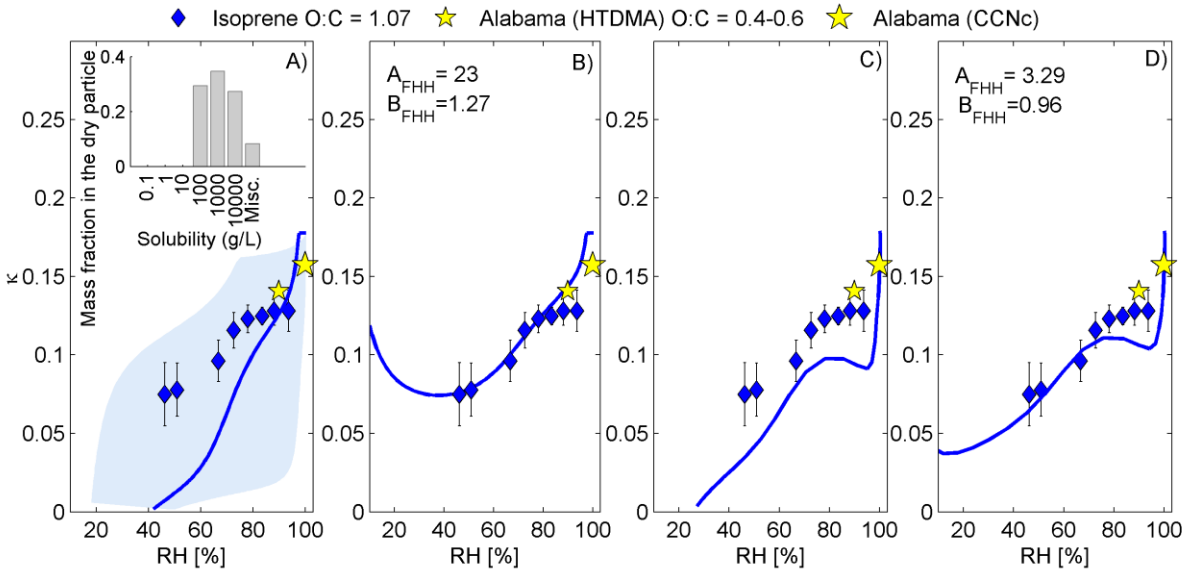

Figure S5. Comparison of modelled κ values for isoprene IP (blue lines) with experimental data (blue points) and ambient data from Alabama (yellow stars). Model calculations account for A) dissolution, B) dissolution and adsorption, C) dissolution and non-ideality, D) dissolution, adsorption and non-ideality. Note: the subplot in (A) shows the solubility distribution assumed for isoprene (IP). The solubility value assigned for Misc (miscible) bin was 10^5 g L^{-1}. IP with a high average oxygen-to-carbon ratio (O:C = 1.07) has high water solubility, ranging from 100 g L^{-1} to fully soluble. Sensitivity to the predicted solubility distribution is shown by the shaded area; the upper limit represents the results for predicted solubility values multiplied by 10 and the lower limit for solubility values divided by 10, the Misc bin value was kept constant as 10^5 g L^{-1}. The laboratory and field data were collected at room temperature. The theoretical calculations were conducted for 298 K.

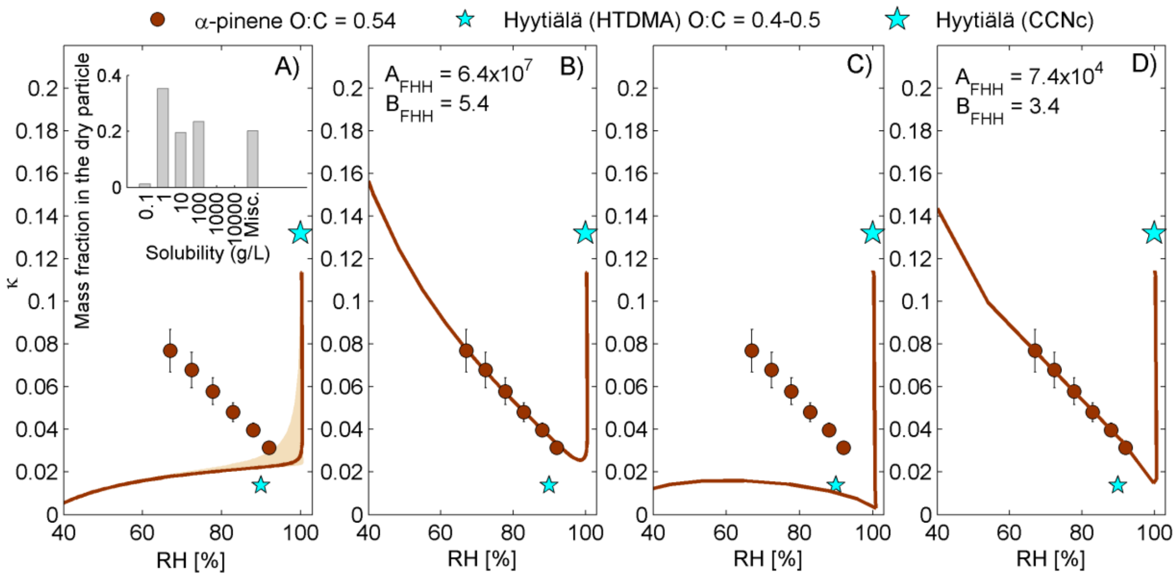

Figure S6. Comparison between modelled κ values for monoterpene MT (red lines) and experimental data (red points) and ambient data from Hyytiälä (blue stars). Model calculations account for A) dissolution, B) dissolution and adsorption, C) dissolution and non-ideality, D) dissolution, adsorption and non-ideality. Note: the subplot in (A) shows the solubility distribution assumed for monoterpene (MT). The solubility value for Misc (miscible) bin was 10^5 g L^{-1}. MT with (O:C = 0.54) is less soluble in water than IP with values mainly between 0.1 and 100 g L^{-1} and about 20% of mass in the Misc bin. Sensitivity to the predicted solubility distribution is shown via the shaded area; the upper limit represents the results for predicted solubility values multiplied by 10 and the lower limit for solubility values divided by 10, and the Misc bin value was kept constant as 10^5 g L^{-1}. The laboratory and field data were collected at room temperature. The theoretical calculations were conducted for 298 K. The theoretical calculations were conducted for 298 K.

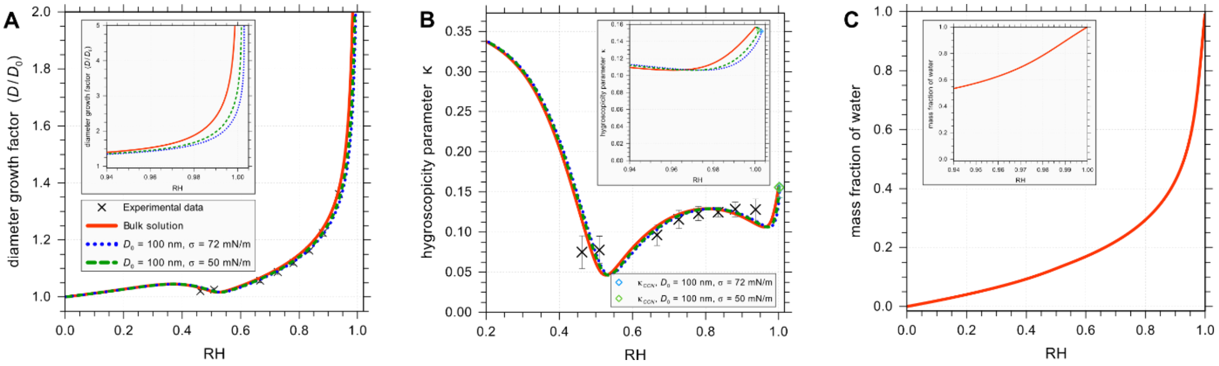

Figure S7. Predicted hygroscopic growth properties of the system of isoprene photo-oxidation products listed in Table S2. Model calculations at 298.15 K are based on AIOMFAC (activity coefficients, non-ideality) and pure component vapor pressures predicted by EVAPORATION. (**A**) Computed hygroscopic growth factor *vs.* RH for the bulk mixture and for particles of dry diameter D_0 = 100 nm with different values (72 mN m^{-1} or 50 mN m^{-1}) assumed for the air-particle surface tension, σ. (**B**) Predicted effective κ_{HGF} for the bulk solution and different particles as in (A). The blue and green diamonds show the predicted critical values for CCN activation, κ_{CCN}. (**C**) Mass fraction of water in the liquid (particle) mixture. The insets show details of the model predictions at high RH. Experimental data (crosses) are from Pajunoja et al. (2015)[23]. The calculations include consideration of a potential liquid-liquid phase separation (not occurring here) and equilibrium gas-particle partitioning of water. Equilibration of semi-volatile organic compounds was constrained by an adjusted vapor pressure based on RH-dependent bounced fraction measurements as described in Section 3.2.

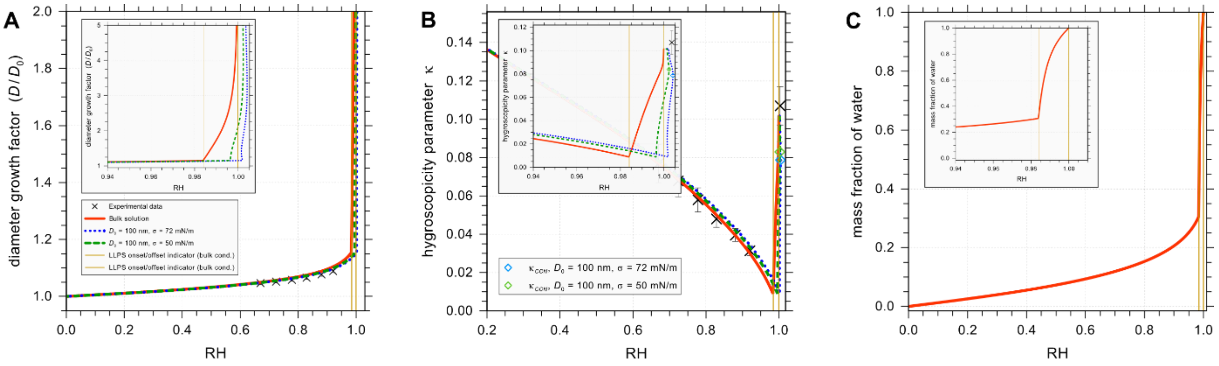

Figure S8. Predicted hygroscopic growth properties of the system of monoterpene ozonolysis products listed in Table S3 (including an Ester dimer compound formed from the reaction of pinic acid and hydroxypinonic acid). Model calculations at 298.15 K are based on AIOMFAC (activity coefficients, non-ideality) and pure component vapor pressures predicted by EVAPORATION. (**A**) Computed hygroscopic growth factor *vs.* RH for the bulk mixture and for particles of dry diameter D_0 = 100 nm with different values (72 mN m^{-1} or 50 mN m^{-1}) assumed for the air-particle surface tension, σ. (**B**) Predicted effective κ_{HGF} for the bulk solution and different particles as in (A). The blue and green diamonds show the predicted critical values for CCN activation, κ_{CCN}. (**C**) Mass fraction of water in the liquid (particle) mixture. The insets show details of the model predictions at high RH. Experimental data (crosses) are from Pajunoja et al. (2015). The calculations include consideration of a potential liquid-liquid phase separation (occurring here in a small range at high RH) and equilibrium gas-particle partitioning of water. Equilibration of semi-volatile organic compounds was constrained by an adjusted vapor pressure based on RH-dependent bounced fraction measurements as described in Section 3.2.

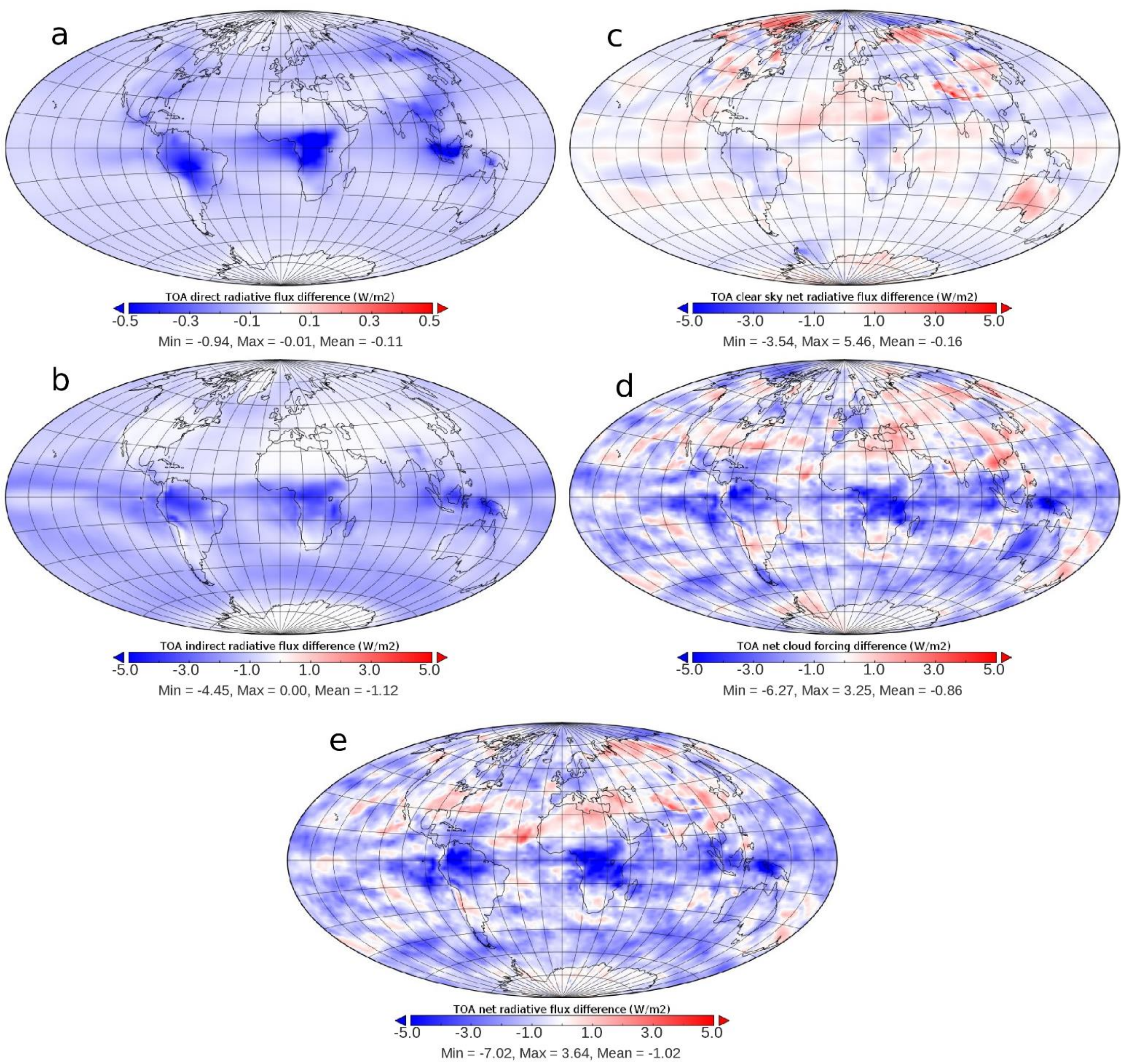

Figure S9. A) Direct (global mean of -0.11 Wm^{-2}) B) Indirect (global mean of -1.12 W m^{-2}) radiative forcing at the top of the atmosphere presented for present day (PD) and offline mode (aerosols are not allowed to affect meteorology). C) FSNTC (Clear Sky Net Solar Flux at Top of the Atmosphere) with a global mean value of -0.26 Wm^{-2}. D) SWCF (Short Wave Cloud Forcing) with a global mean value of -0.86 Wm^{-2}. E) FSNT (Net solar flux at Top of the Atmosphere) with a global mean value of -1.20 Wm^{-2}. Presented for present day (PD) and online mode (aerosols are allowed to affect meteorology). All panels show the difference between the case with κ_{OA}= 0.15 (high case) and κ_{OA} = 0.05 (low case), i.e. high case minus low case. These results are from simulated with NorESM1, see Table S4.

Table S1. Experimental conditions and separation relative humidity (SRH) for SOA produced by isoprene photo-oxidation. Particles were collected on hydrophobic substrates using an electrostatic precipitator and single stage impactor. SRH = 0 indicates no LLPS observed during humidity cycles.

SOM sample	VOC conc. (ppm)	O_3 conc. (ppm)	SOM mass conc. (μg m^{-3})	Flow rate for SOM particle production (L m^{-1})	Collection time (hour)	Collection method	SRH (%)	
							drying	moistening
Isoprene 1	0.7	30	60-70	7.0	24	Single stage impactor	0	0
Isoprene 2	0.7	30	60-70	7.0	22	Single stage impactor	0	0
Isoprene 3	4	10	100-200	9.5	72	Electrostatic precipitator	0	0
Isoprene 4	7	10	300-400	9.5	48	Electrostatic precipitator	0	0
Isoprene 5	7	13	500-1000	7.0	96	Electrostatic precipitator	0	0
Isoprene 6	7	13	500-1000	7.0	96	Electrostatic precipitator	0	0
Isoprene 7	7	13	500-1000	7.0	168	Electrostatic precipitator	0	0
Isoprene 8	7	13	500-1000	7.0	168	Electrostatic precipitator	0	0

Table S2. Properties of isoprene (IP) photo-oxidation products used for the model predictions.

MCM species	Mass fraction [(a)]	Molar amount [(b)] (nmol m^{-3})	Molecular structure (SMILES)	*M* (g mol^{-1})	Solubility (g L^{-1})	ρ (kg m^{-3})
IEB1OOH	0.308	29.544	OCC(O)C(C)(OO)C=O	150	4850	1500
IEB2OOH	0.254	2.282	OOC(C=O)C(C)(O)CO	150	5150	1500
C59OOH	0.212	38.675	OCC(=O)C(C)(CO)OO	150	92.6	1470
IEC1OOH	0.074	13.500	OCC(=O)C(C)(CO)OO	150	92.6	1470
C58OOH	0.032	3.069	O=CC(O)C(C)(CO)OO	150	Miscible	1530
IEPOXA	0.030	0.000	CC(O)(CO)C1CO1	118	Miscible	1180
C57OOH	0.026	2.494	OCC(O)C(C)(OO)C=O	150	4850	1500
IEPOXC	0.013	0.000	CC1(CO1)C(O)CO	118	1390	1150
HIEB1OOH	0.008	2.390	OCC(O)C(CO)(OO)C=O	166	7140	1730
INDOOH	0.007	1.749	OCC(ON(=O)=O)C(C)(CO)OO	197	Miscible	1690
IEACO3H	0.006	0.000	CC(O)(C1CO1)C(=O)OO	148	226	1430
C525OOH	0.006	1.796	OCC(=O)C(CO)(CO)OO	166	Miscible	1680
HIEB2OOH	0.004	1.178	OOC(C=O)C(O)(CO)CO	166	7350	1710
IEC2OOH	0.004	0.000	OCC(=O)C(C)(OO)C=O	148	5500	1390
INAOOH	0.004	0.971	OCC(C)(OO)C(O)CON(=O)=O	197	Miscible	1650
C510OOH	0.003	0.029	O=CC(O)C(C)(OO)CON(=O)=O	195	8640	1630
INB1OOH	0.002	0.500	OCC(OO)C(C)(CO)ON(=O)=O	197	Miscible	1690
IECCO3H	0.002	0.000	CC1(CO1)C(O)C(=O)OO	148	151	1410
INCOOH	0.001	0.215	OCC(OO)C(C)(O)CON(=O)=O	197	Miscible	1660
INB2OOH	0.001	0.244	OOCC(O)C(C)(CO)ON(=O)=O	197	Miscible	1650
Tetrol dimer [(c)]	-	39.109	CC(O)(CO)C(O)COC(C)(CO)C(O)CO	254	-	1181

[(a)] Particle-phase abundance in terms of compound mass fractions according to the MCM(v3.2)-SIMPOL simulation conducted by Chen et al. (2011) for RH = 40 %, T = 298 K, and an organic particle mass concentration of 3.6 μg m^{-3} of air.

[(b)] Total molar amounts (gas + particle phase) of the organic components per m^3 of air as determined by the gas-particle partitioning model based on AIOMFAC + EVAPORATION when evaporative loss during the drying stage of the corresponding experiments is accounted for. These amounts yield an organic particle mass concentration of ~20 μg m^{-3}, comparable to the conditions for the HTDMA experiments with the IP system. The listed densities are also from the online version of the SPARC property estimator.

[(c)] A Tetrol dimer compound, typically found in isoprene SOA under low-NO_x conditions *[Surratt et al., 2011]*, was introduced for the AIOMFAC + EVAPORATION model to produce a particle phase mixture with an average O:C ratio that matches the laboratory experiments.

Table S3. Properties of monoterpene (MT) ozonolysis products used for the model predictions.

MCM species	Mass fraction [(a)]	Molar amount [(b)] (nmol m^{-3})	Molar amount [(c)] (nmol m^{-3})	Molecular structure (SMILES)	M (g mol^{-1})	Solubility (g L^{-1})	ρ (kg m^{-3})
PINIC	0.226	56.777	30.091	OC(=O)CC1CC(C(=O)O)C1(C)C	186	4.5	1140
C812OH	0.138	37.423	37.423	OCC1CC(O)(C(=O)O)C1(C)C	174	79.7	1200
HOPINONIC	0.126	26.956	0.270	OCC(=O)C1CC(CC(=O)O)C1(C)C	200	3.54	1150
C813OH	0.091	24.231	24.231	OCC(CC(=O)C(=O)O)C(C)(C)O	190	99.5	1320
C921OOH	0.084	21.218	21.218	OCC(=O)C1(OO)CC(CO)C1(C)C	204	15.1	1260
C813OOH	0.080	22.178	22.178	OCC(CC(=O)C(=O)O)C(C)(C)OO	206	18.5	1400
C922OOH	0.071	18.378	18.378	OCC(=O)C(=O)CC(CO)C(C)(C)OO	220	Miscible	1360
C108OOH	0.044	0.424	0.424	O=CCC(CC(=O)C(=O)C)C(C)(C)OO	216	Miscible	1210
C108OH	0.028	0.001	0.001	O=CCC(CC(=O)C(=O)C)C(C)(C)O	200	Miscible	1140
C98OOH	0.025	4.835	4.835	OCC(CC(=O)C(=O)C)C(C)(C)OO	204	Miscible	1240
C98OH	0.017	0.068	0.068	OCC(CC(=O)C(=O)C)C(C)(C)O	188	Miscible	1170
C812OOH	0.011	3.002	3.002	OCC1CC(OO)(C(=O)O)C1(C)C	190	0.452	1270
C107OH	0.009	0.000	0.000	O=CCC1CC(O)(C(=O)C)C1(C)C	184	Miscible	1050
C813O2	0.008	0.000	0.000	OCC(CC(=O)C(=O)O)C(C)(C)O[O]	205	18.5	1400
C922O2	0.007	0.000	0.000	OCC(=O)C(=O)CC(CO)C(C)(C)O[O]	219	Miscible	1360
C97OOH	0.007	0.448	0.448	OCC1CC(OO)(C(=O)C)C1(C)C	188	13.8	1140
C811PAN	0.005	0.044	0.044	O=N(=O)OOC(=O)CC1CC(C(=O)O)C 1(C)C	247	6.15	1300
C97OH	0.005	0.001	0.001	OCC1CC(O)(C(=O)C)C1(C)C	172	62.9	1080
C89CO2H	0.004	0.000	0.000	O=CCC1CC(C(=O)O)C1(C)C	170	45.6	1060
C920PAN	0.003	0.038	0.038	OCC(=O)C1CC(CC(=O)OON(=O)=O) C1(C)C	261	10.5	1300
C811OH	0.002	0.024	0.024	OCC1CC(C(=O)O)C1(C)C	158	12.7	1090
PINONIC	0.002	0.000	0.000	OC(=O)CC1CC(C(=O)C)C1(C)C	184	26.2	1040
Ester dimer	0.0	0.000	26.687		368	?	1105

[(a)] Particle-phase abundance according to the MCM-SIMPOL simulation conducted by Chen et al. (2011) for RH = 40 %, T = 298 K, and an organic particle mass concentration M_{org} = 3.6 µg m^{-3}.

[(b)] Total molar amounts (gas + particle phase) of the organic components per m^3 of air as determined by the gas-particle partitioning model based on AIOMFAC + EVAPORATION when evaporative loss during the drying stage of the corresponding experiments is accounted for. These amounts yield an organic particle mass concentration of ~40 µg m^{-3}, comparable to the conditions for the HTDMA experiments with the MT system.

[(c)] System with species as in (b), but with an additional Ester dimer compound which is formed from the reaction of PINIC and HOPINONIC, limited by consuming ~99 % (by molar amount) of HOPINONIC initially predicted by MCM.

Table S4. List of NorESM simulations.

Simulation	Simulation period	Simulation length	Organic aerosol κ	Aerosol-meteorology interaction
1	Present-day (2000)	7 years (Last 5 years analysed)	0.01	Off
2	Present-day (2000)	7 years (Last 5 years analysed)	0.05	Off
3	Present-day (2000)	7 years (Last 5 years analysed)	0.1	Off
4	Present-day (2000)	7 years (Last 5 years analysed)	0.15	Off
5	Present-day (2000)	22 years (Last 15 years analysed)	0.01	On
6	Present-day (2000)	22 years (Last 15 years analysed)	0.05	On
7	Present-day (2000)	22 years (Last 15 years analysed)	0.1	On
8	Present-day (2000)	22 years (Last 15 years analysed)	0.15	On
